# Reactive molecular dynamics simulations of lithium-ion battery electrolyte degradation

**DOI:** 10.1038/s41598-024-60063-0

**Published:** 2024-05-04

**Authors:** Y. Mabrouk, N. Safaei, F. Hanke, J. M. Carlsson, D. Diddens, A. Heuer

**Affiliations:** 1 Forschungszentrum Jülich GmbH, Helmholtz-Institute Münster (IEK-12), Corrensstraße 46, 48149 Münster, Germany; 2 Dassault Systémes Deutschland GmbH, Am Kabellager 11-13, 51063 Cologne, Germany; 3 Dassault Systémes, Cambridge, CB4 0WN UK; 4 Institute of Physical Chemistry, University of Münster, Corrensstrasse 28/30, 48149 Münster, Germany

**Keywords:** Batteries, Chemical physics

## Abstract

The development of reliable computational methods for novel battery materials has become essential due to the recently intensified research efforts on more sustainable energy storage materials. Here, we use a recently developed framework allowing to consistently incorporate quantum-mechanical activation barriers to classical molecular dynamics simulations to study the reductive solvent decomposition and formation of the solid electrolyte interphase for a graphite/carbonate electrolyte interface. We focus on deriving condensed-phase effective rates based on the elementary gas-phase reduction and decomposition energy barriers. After a short initial transient limited by the elementary barriers, we observe that the effective rate shows a transition to a kinetically slow regime influenced by the changing coordination environment and the ionic fluxes between the bulk electrolyte and the interface. We also discuss the impact of the decomposition on the ionic mobility. Thus, our work shows how elementary first-principles properties can be mechanistically leveraged to provide fundamental insights into electrochemical stability of battery electrolytes.

## Introduction

The development of predictive simulation frameworks for novel battery electrolytes is of special interest due to the recently increased use of rechargeable batteries^1–4^. Such frameworks hold the promise for advancing battery innovation by providing mechanistic insights into the working principles underlying the functionality of electrochemically stable and ionically conductive electrolytes. For example it would ideally be possible to rationally design optimal electrolyte formulations based on film-forming additives and high-quality ionic conductors. While this objective has arguably been achieved for specific subsets of electrolyte properties, such as those involving bulk transport and conduction phenomena^1,2,4^, the prediction of interfacial properties seems to be more challenging^5–8^. More specifically, classical molecular dynamics (MD) simulations are established as an effective framework for predicting bulk electrolyte conductivity^1–3^ but they are inherently limited to bulk systems since they do not account for chemical or electrochemical changes taking place at the materials interfaces. Ab-initio molecular dynamics (AIMD) are in principle well-suited for this task but they have a major limitation with respect to the accessible simulation time-scale^9–11^. It is therefore of interest to seek an efficient, physically motivated and simple framework to incorporate chemical reactions to classical MD simulations^12,13^. The aim of this work is to demonstrate the use of the reactive step MD (rs@md)^14,15^ framework in the context of modeling electrolyte decomposition and degradation reactions occurring at the anode-electrolyte interface of Lithium-ion battery systems (LIBs)^1,2^. rs@md is a framework aimed for enabling the occurrence of bond-breaking and formation within classical MD simulations by means of time-dependent modifications of classical force-fields. This framework was introduced in previous work^14,15^ and benchmarked to AIMD calculations with respect to the kinetic information at short time-scales for simple systems^15^. It is therefore of interest to explore the use of rs@md for longer time-scales and more realistic model systems, in particular whether and how rs@md can be used to identify connections between atomic simulations and macroscopic continuum and lattice models^7,8^. The main aspects discussed in this work are the formation kinetics of the solid electrolyte interphase (SEI)^1–3^ for a graphite/carbonate electrolyte model system as well as the the effect of reactions on the structural and dynamical properties of the baseline electrolyte.

Despite the wide availability of both characterization and electrochemical performance data related to the SEI, quantitatively correlating the baseline electrolyte chemistry with key performance indicators is known to be a challenging task. Well-known examples of experimentally fairly established yet quantitatively not explained trends include the sensitivity of cycling performance with respect to specific electrolyte additives such as fluorethylene carbonate (FEC) or vinylene carbonate (VC), the dependence of the capacity fading rate on temperature^16,17^ and on the state of charge^18^, the dependence of the first-cycle irreversible capacity on the active surface area^19–22^, and the sensitivity of electrochemical properties to the crystal orientation and the crystallinity of the electrode^19,23,24^. But instances where these trends have been rigorously addressed within a first-principles computational framework are rare^22^, and a mechanistic framework allowing the systematic derivation of these experimental results is still lacking. Thus, SEI formation provides an overall ideal case study for the exploration and critical assessment of the predictions that can be made on the basis of our methods. In this work, we use rs@md to map gas-phase energy barriers of SEI formation reactions to liquid phase reaction rates. This mapping is particularly motivated by the importance of medium effects on bimolecular nucleophilic substitution reactions involving ions and protic solvents^10,11,25^. We show that the effective formation rates transition from an activation limited reaction rate to a diffusion and electro-migration limited reaction rate within a time-scale of few $\sim \text{ns}$. In particular, we focus on the effect of the gas-phase reaction barriers of the initial charge transfer reaction and show that charge transfer rates within the order of $\sim \text{ps}^{-1}$ imply the onset of electro-migration and diffusion limited reaction rate within a time-scale of few $\sim \text{ns}$. Furthermore, we show that the formation of coordinating structures of Li$^{+}$ and negatively charged ethylene carbonate (EC) radicals is effectively a limiting factor in the initial stage of SEI growth reactions.

The outline of the manuscript is as follows. In the “Methods” section we provide a detailed description of the simulation framework and the underlying model. The subsequent “Results” section contains a discussion of the baseline system without the presence of chemical reactions. In the next step the impact of the reaction kinetics is presented. Subsequently the resulting structural changes of the baseline electrolyte after decomposition will be outlined. Finally, the results are discussed.

## Methods

### Simulation framework

**Figure 1:**
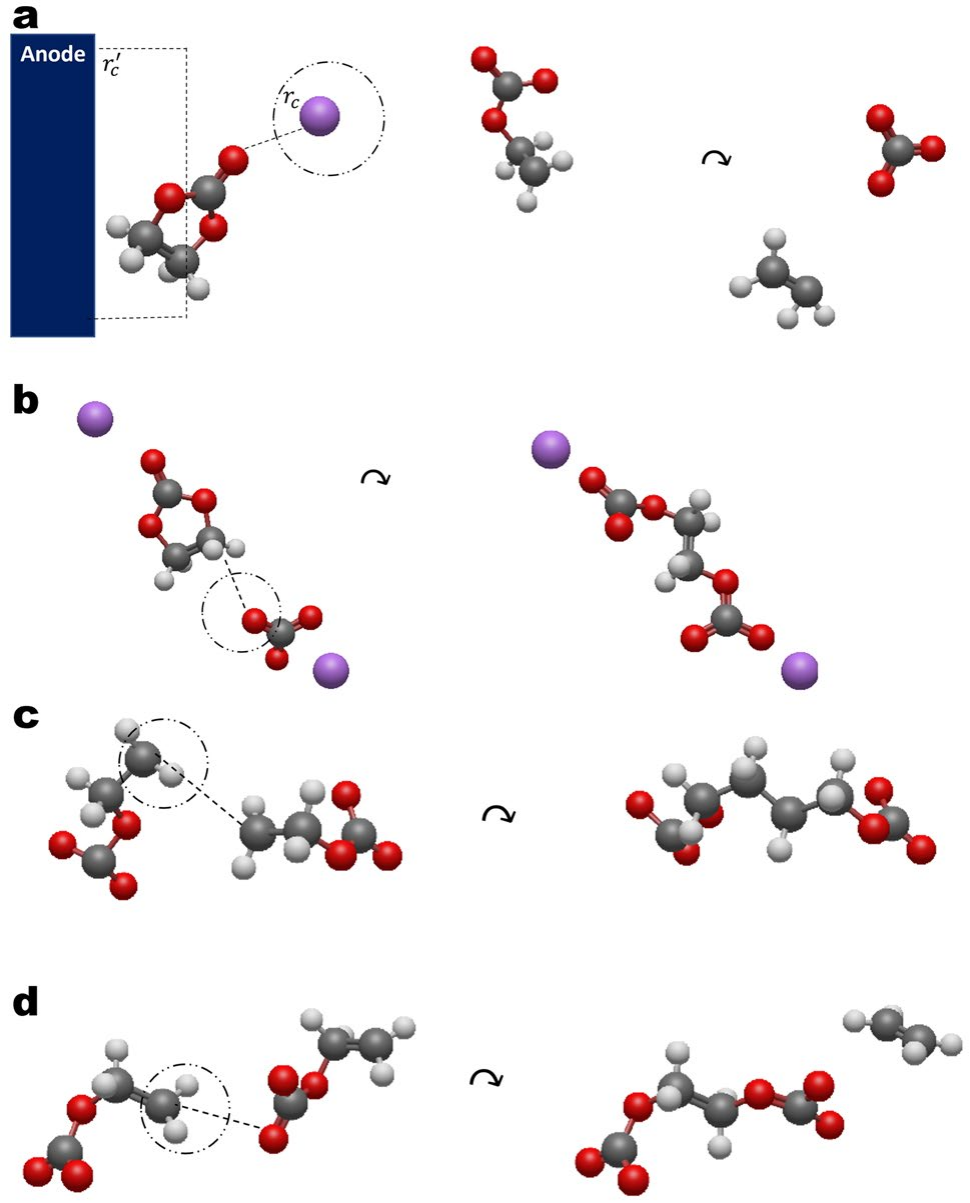
Coordination criteria for the reaction network. The pathway is based on^13^ and earlier related works^25^. (**a**) For the first reduction of EC one Li$^{+}$ must be coordinated to the carbonate group (the distance measured with respect to the double bonded oxygen) and the EC must be within 1 nm from the contact plane. (**b**) For the formation of EDC$^{2-}$ the carbonate anion must coordinate to the ethene group with two additional Li$^{+}$ coordinations. (**c**) For the formation of BDC$^{2-}$ the criterion is the distance between the two carbons of the ethene group of oEC$^{-}$ (in addition to two coordinated Li$^{+}$). (**d**) The second path for EDC$^{2-}$ formation is the coordination of the ethene carbon to the double bonded oxygen of the oEC$^{-}$ (in addition to two coordinated Lithium ions). In all cases (**a**–**d**) the threshold distance is set to the first minimum of the RDF of the corresponding species.

rs@md consists of performing a classic MD simulation that is periodically interrupted in order to insert or remove molecular bonds according to the definitions of the involved reactions^13–15^. The conditions for the insertion or removal of bonds can be subdivided into geometric and dynamic conditions. In the former the spatial proximity of the reactants is verified while the latter corresponds to the probability of a reaction to occur per unit time given that the first condition is met. The geometric conditions (depicted in Fig. 1) are derived from classic force field parameters and correspond to inter-molecular cut-off distances while the dynamic conditions are defined via the “gas-phase” energy difference^13^ between the initial state and the transition state of the reactive complex in accordance with transition state theory^13–15^. The operation of bond rearrangement has previously been shown to involve non-trivial difficulties related with the smoothness of the resulting force field^14^ and an approach based on performing local relaxations of the product molecules after each reaction has consequently been suggested^14^. The consistency of this approach with respect to structural and dynamic system properties has been verified^15^ by means of a comparison with AIMD simulations of dimerization of singly reduced EC radicals in solution to form dilithium butyl dicarbonate (Li$_{2}$BDC). Similar approaches were used to include electrochemical charge-transfer reactions in an electrode/electrolyte interface leading to SEI formation^12,13^ and showed that a porous passivation layer developed within simulation-times of $\sim 10\; \text{ns}$. The present work focuses on the effect of elementary charge transfer rates on the effective formation rates. The force field used for the MD runs is OPLS-AA optimized for conformational and energetic properties of organic liquids^26^ and the CL & P force field for ionic liquids^27^. The simulations were performed with the GROMACS-2019.3 version^28^. The initial structures of the electrolyte were generated with the open-source software PACKMOL^29^. All results described above have been cross-validated using the methods described in Abbot and Hanke^13^ using the COMPASSIII forcefield^30^ with systems constructed with the Materials Studio Amorphous cell module^31^.

### Model system

A three-dimensional frozen graphitic anode structure in contact with a carbonate electrolyte composed of lithium hexa-fluorophosphate (LiPF$_{6}$) salt dissolved in a pure EC solution is implemented as the baseline system. Throughout all simulations the initial electrolyte structures have been generated by randomly packing 140 LiPF$_{6}$ pairs and 1800 EC molecules within a rectangular cell of $3.5\times 3.5\times 17 \, [\text{nm}^{3}]$. The ratio of salt to solvent molecules corresponds to the one molar reference value of the salt concentration^12,13^. Previous work has established that most of the electrolyte decomposition products originate from the EC solvent molecule due to its high polarity and strong interaction with the electrode surface. Therefore this justifies the simplified choice of EC as the only solvent molecule in the reactive model^12,13^. The implemented pathway consists of a sequence of fragmentation and dimerization reactions initiated by the electron transfer from the graphite electrode to the EC molecule. This mechanism is supported by ample evidence and numerous investigations from both the experimental and theoretical communities^32–35^. EC is not expected to be stable under standard operation conditions because the lowest unoccupied molecular orbital (LUMO) of EC was shown to lie below the Fermi level of lithiated graphite^8^. The decomposition peak of EC is correspondingly observed around $1\; \text{V}$ at the Li/Li$^{+}$ scale, while the voltage operation range of the graphite anode is close to $0.1\; \text{V}$^8^. The decomposition products of the implemented pathway (Li$_{2}$CO$_{3}$ and Li$_{2}$EDC/Li$_{2}$BDC) are also widely reported from characterization studies based on Fourier transform infrared-red spectroscopy (FTIR)^36^ as well as X-ray photo-electron spectroscopy (XPS)^32^. The pathway is summarized in Fig. 1 and the related details can be found in our former work^13^.

**Figure 2:**
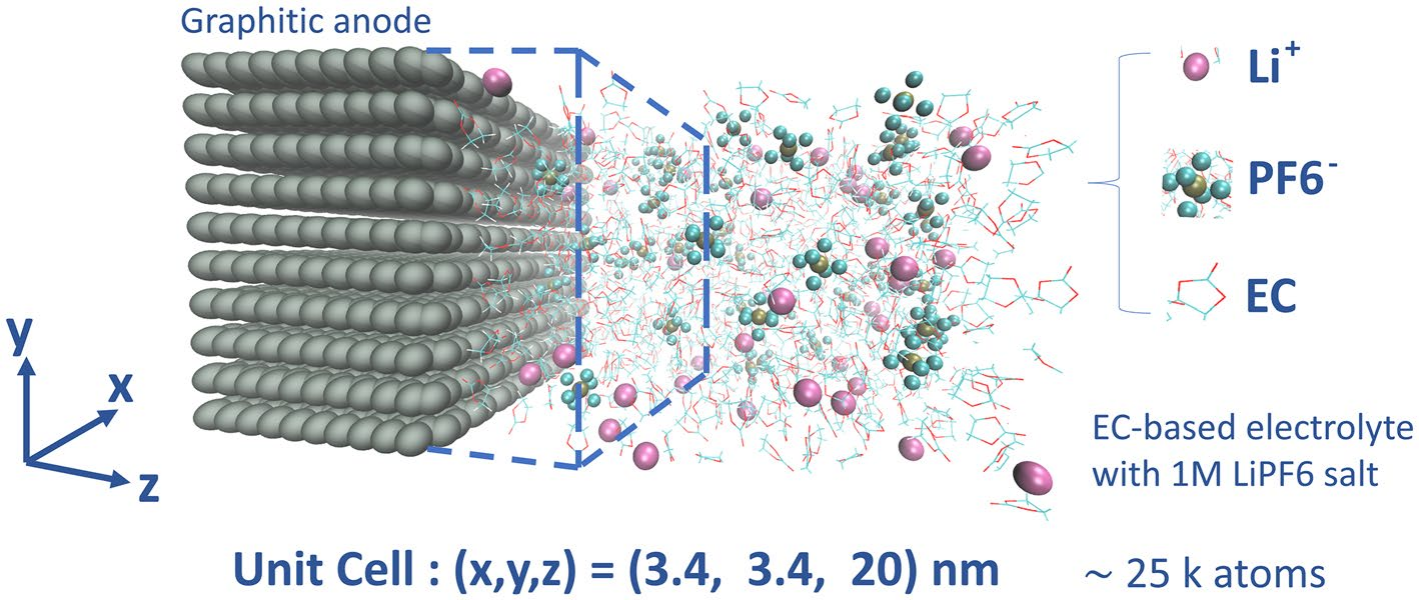
Three-dimensional representation of the slab model. The critical distance from the electrode for the occurrence of reduction reactions is indicated with a dashed blue line. In this representation the cell extension along the $z$-direction is reduced for visual convenience.

The slab model is depicted in Fig. 2. The slab geometry is implemented by imposing periodic boundary conditions (PBC) along all three coordinates. The choice of PBC along the $z$-coordinate allows using the graphite structure as an effective wall from the two ends of the electrolyte phase. A negative electric charge of $-1.25\times 10^{-2}\, e$ is assigned to the carbon atoms at the graphite/electrolyte contact surface implying a total surface charge of $-2\, e$ homogeneously distributed over the $3.5\times 3.5$ [$\text{nm}^{2}$] surface, and thus a charge density of $\sigma =3.1 \frac{\upmu \text{C}}{{\text{cm}}^{2}}$^12,13^. This value implies a voltage difference in the order of $\sim 1\; \text{V}$ between the electrode and the electrolyte if a charged infinite plane model is assumed for the potential drop between the electrode and the electrolyte^12,13^. The excess surface charge is compensated with a uniform positive background to ensure electroneutrality. The restriction to a uniform fixed charge on the electrode is justified as the electrode response to local charge fluctuations is not expected to significantly affect the results^37–39^. The cut-off scheme used for the MD-integration consists of the Verlet algorithm within a discretization time of $1 \; \text{fs}$. The non-bonded Coulomb forces were computed based on the particle mesh Ewald summation scheme (PME) with a Coulomb cut-off of 1.6 [nm]^28^. Charge neutrality is imposed by a particle injection scheme to compensate the charger transfer reactions (see Supplementary Information). The temperature coupling algorithm used is the Nose-Hoover method^28^. Unless otherwise specified the reference temperature was set to $T=300\; \text{K}$. The hydrogen bonds were constrained based on the LINCS algorithm^28^. Based on an activation energy in units of kJ/mol the reaction rate in units of time is defined as $\nu =\frac{k_{B}T}{h}\times e^{-\frac{E_{a}}{k_{B}T}}$. The reaction probability is defined as the product of the reaction rate with the length of the MD-run. Assuming a barrierless reaction with $E_{a}=0$ the highest rate following the definition would be $6.12\; \text{ps}^{-1}$. The time between two reaction steps is set to $\tau =10^{-1} \; \text{ps}$ in order to have a reaction probability smaller than unity. In total, $N=10^{5}$ reaction steps are performed, corresponding to a total run time of the MD simulations of $\sim 10\; \text{ns}$. The input values used for the reaction barriers $E_{a}$ are derived from our previous work^13^ based on DFT-optimizations of solvation configurations and a brief summary and comparison to the experimentally known reduction potential of EC are given in the Supplementary Information.

## Results

### Pre-reactive simulations

**Figure 3:**
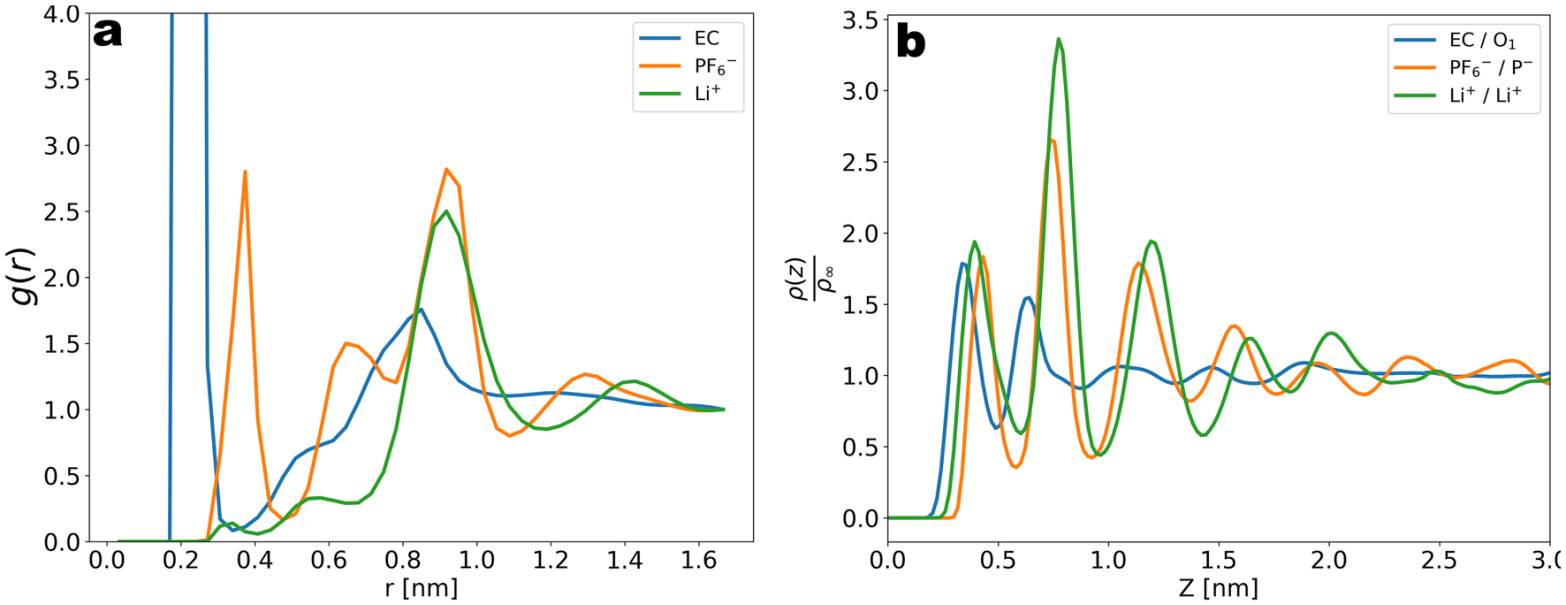
(**a**) Radial distribution function (RDF) of Li$^{+}$/EC(O), Li$^{+}$/PF6$^{-}$(P) and Li$^{+}$/Li$^{+}$. (**b**) Density distribution along the slab direction for Li$^{+}$, PF6$^{-}$ and EC. The excess of Li$^{+}$ within $z = 3 \; \text{nm}$ corresponds to the negative surface charge on the graphite surface.

We first consider the radial distribution function (RDF) of the Li$^{+}$ cations with the PF6$^{-}$ anions and the EC molecule. The reference point for the EC/Li$^{+}$ RDF is defined as the double bonded oxygen, and for the PF6$^{-}$/Li$^{+}$ RDF the reference point is defined as the phosphorus atom. In the two cases we recognize in Fig. 3a a well-defined first peak going from about 0.2 nm to 0.35 nm before the saturation. The area under the first peak of the EC(O)/Li$^{+}$ RDF results in a coordination number equal to 4.1 in agreement with the literature^40,41^. This large value reflects the solvation of Li$^{+}$ by 4 EC molecules and is a consequence of using a high polarity molecule as the only solvent, while the lower height of the first peak in the PF6$^{-}$/Li$^{+}$ RDF reflects the well-dissolved structure of the salt. A further important property is the density distribution within the liquid phase along the slab direction. In the density distribution of the three species (EC, Li$^{+}$ and PF6$^{-}$ in Fig. 3b) we observe three well-pronounced peaks starting at the contact plane ($z=0$) and decaying to a constant value corresponding to the bulk electrolyte density around a distance of $z=3\; \text{nm}$ from the contact plane. The bulk density is estimated to a value of 1.38 g/cm$^{3}$ and is as expected slightly above the reference value 1.32 g/cm$^{3}$ of the pure solvent^42^. The observed peaks reflect the ordered layers of the liquid phase and are qualitatively in line with the familiar structure of the solid-liquid interphase density distribution^43–45^. The excess of Li$^{+}$ compared to PF6$^{-}$ within $z=3\; \text{nm}$ is explained by the negative surface charge of the carbon atoms and is reminiscent of the electric double layer (EDL) structure^46–49^. The overlap seen in the Li$^{+}$ and PF6$^{-}$ peaks might indicate formation of ionic pairs at the interface and is in qualitative agreement with observations in^50^. The charge within $z=3\; \text{nm}$ is calculated from the difference in area under the Li$^{+}$ and PF6$^{-}$ distributions and corresponds to $+1.9\, e$, thus effectively screening the surface charge $-2\, e$ within $z=3\; \text{nm}$. Assuming a surface charge $\sigma =3.1 \; \frac{\upmu {\text{C}}}{\text{cm}^{2}}$ at the graphite is equivalent to an applied voltage of $\phi = \frac{z\sigma }{\epsilon _{0}}\sim 1\; \text{V}$, the resulting capacitance from the charge accumulated in the electrolyte is roughly equivalent to $\frac{\sigma }{\phi }\sim 3.1 \; \frac{\upmu {\text{F}}}{\text{cm}^{2}}$, thus in agreement with the literature^51^.

**Figure 4:**
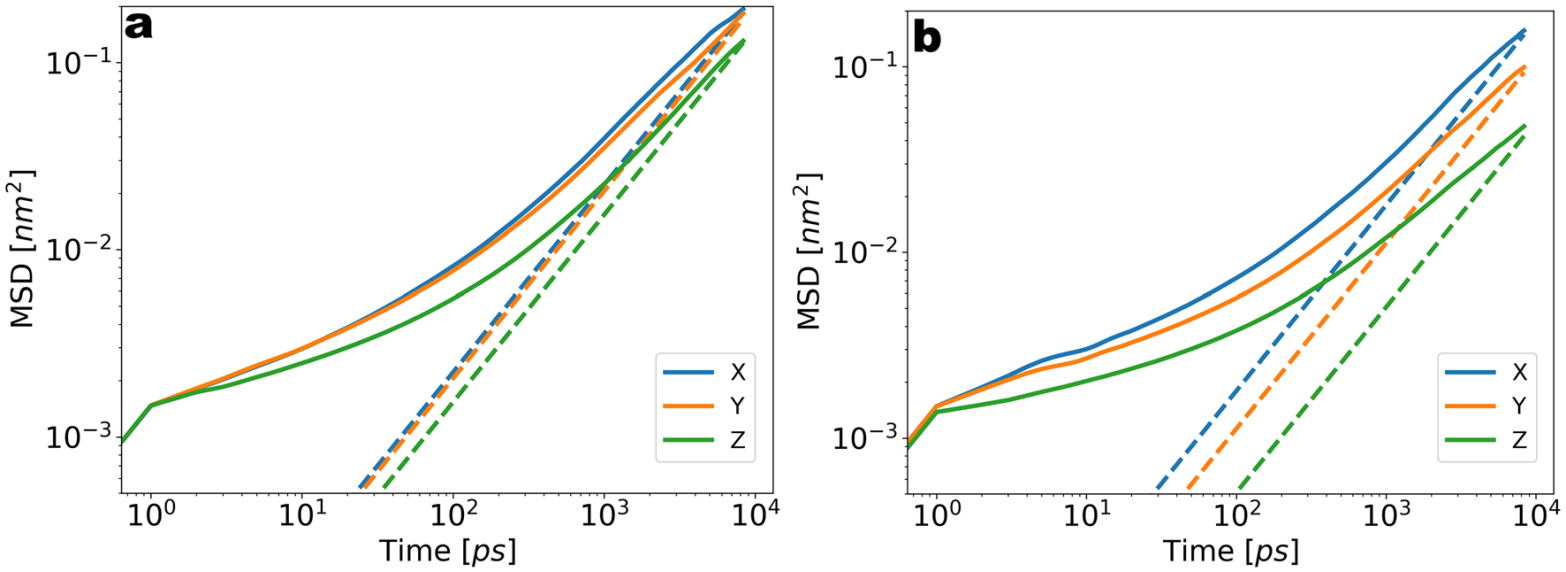
(**a**) Mean squared displacement (MSD) of Li$^{+}$ within $1\; \text{nm}$ from electrode. (**b**) Mean squared displacement (MSD) of Li$^{+}$ at $5 \; \text{nm}$ from electrode. Close to the surface the MSD along *xyz* coordinates is not identical and a drop in the *z*-diffusivity is seen. In both plots the dashed lines represent the linear fits to the MSD in each direction (as upper estimations of the diffusivity due to the lack of strictly linear (diffusive) regime) and the solid lines represent the MSD.

Further we briefly comment on the dynamic properties of the Li$^{+}$ within the electrolyte phase. In order to verify the effect of the density alternations near the contact plane on the diffusivity we calculate the diffusion coefficients from Li$^{+}$ trajectories labeled with respect to the *z*-coordinate. To account for the drift of Li$^{+}$ along the slab direction due to the electrode we subtract the mean displacement from the MSD such that the shift is removed^52^. From the mean squared displacement (MSD) *vs* time plotted in Fig. 4a we recognize a sub-diffusive behaviour indicated by the lack of strictly linear MSD *vs* time relation. An upper estimate of the diffusion coefficient is obtained from the slope of a time-linear function corresponding to the MSD in the last time window, as the onset of the diffusive regime is expected after the simulated time. The deviation from the strictly linear relation is due to the longer time needed for the onset of diffusive scaling due to the presence of the electrode. The estimated slope corresponds to a diffusion constant of $D=5\times 10^{-5} \frac{{\text{nm}}^{2}}{\text{ps}}$ (dashed lines in Fig. 4a) and is in the correct order of magnitude of the reported diffusivity of carbonate electrolytes^53^. In Fig. 4a,b we can see a drop of the diffusivity for small values of *z*, reflecting the effect of the free energy barriers at the solid surface^52^. The drop of diffusivity can be traced to the density alternations due to the layering shown in Fig. 3b. While this effect is most pronounced for the $z$-MSD (a decrease by a factor of 2), the slight anisotropy of the $xy$-MSDs close to the electrode likely reflects the anisotropy of the edge plane of graphite.

### Reactive simulations

**Figure 5:**
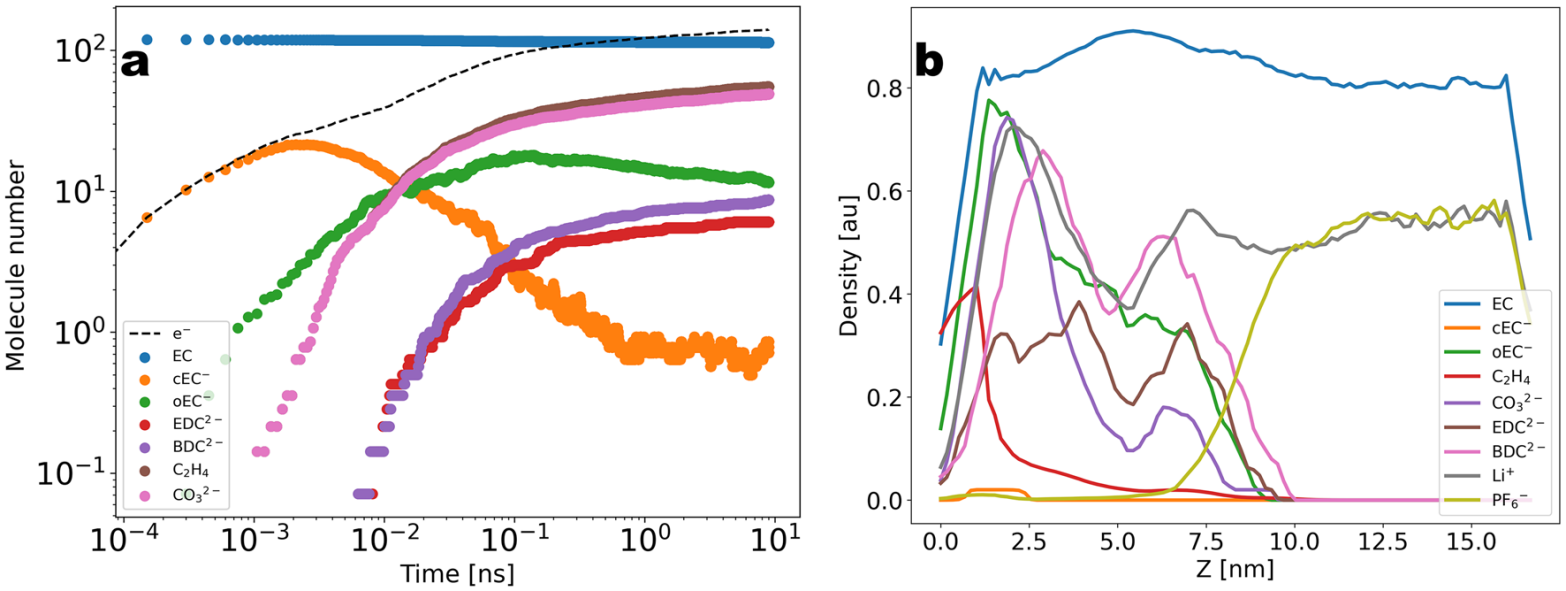
(**a**) Total number of radical-and end-product molecules as a function of time during $T\sim 10\; \text{ns}$ of reactive simulations. The molecule number for EC was re-scaled for visual convenience (in Fig. 9 (**a**) the data is shown in more detail without re-scaling). (**b**) Density distribution along the slab averaged over $T=100\; \text{ns}$ of post-reactive simulations.

**Figure 6:**
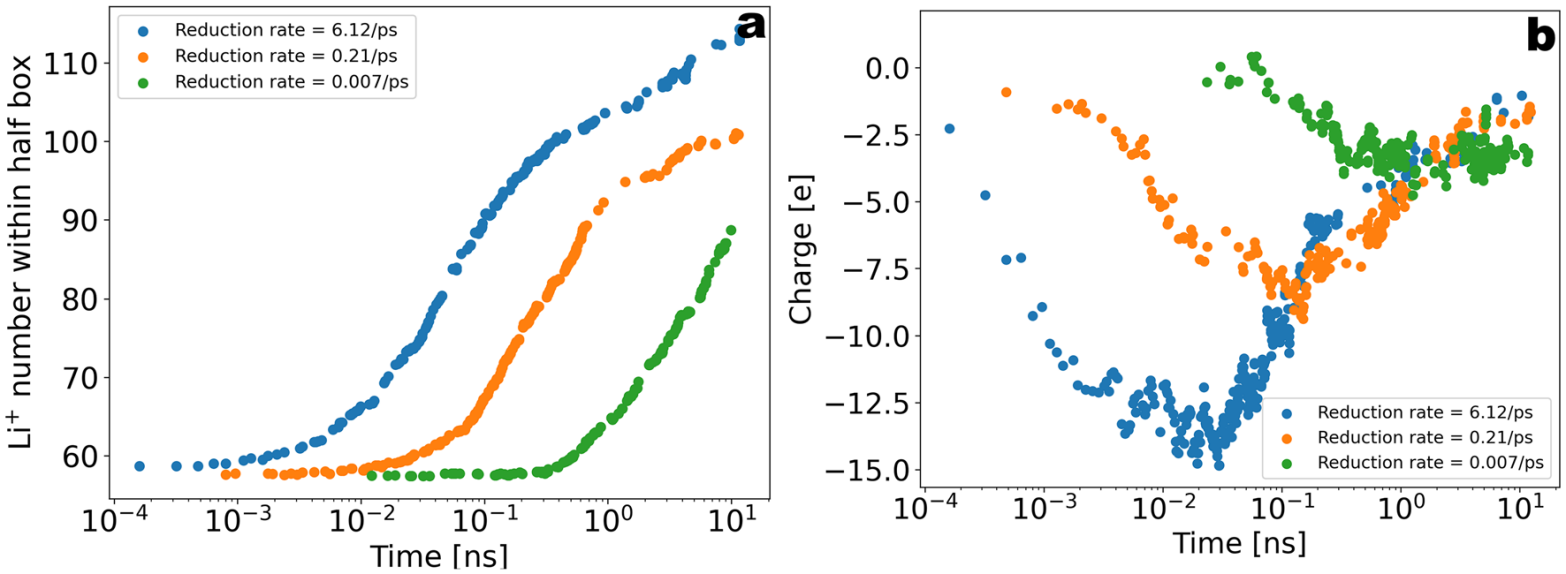
(**a**) Li$^{+}$ number within the half simulation box for the three scanned reduction rates 6.23/ps, 0.021/ps and 0.007/ps. The increase in the Li$^{+}$ number is suggestive for a migration flux due to the reduction reactions. Remarkably, the Li$^{+}$ flux after the first reduction reactions strongly depends on the reduction rate. (**b**) The integrated electric charge within 1nm from the electrode for the three different rates.

**Figure 7:**
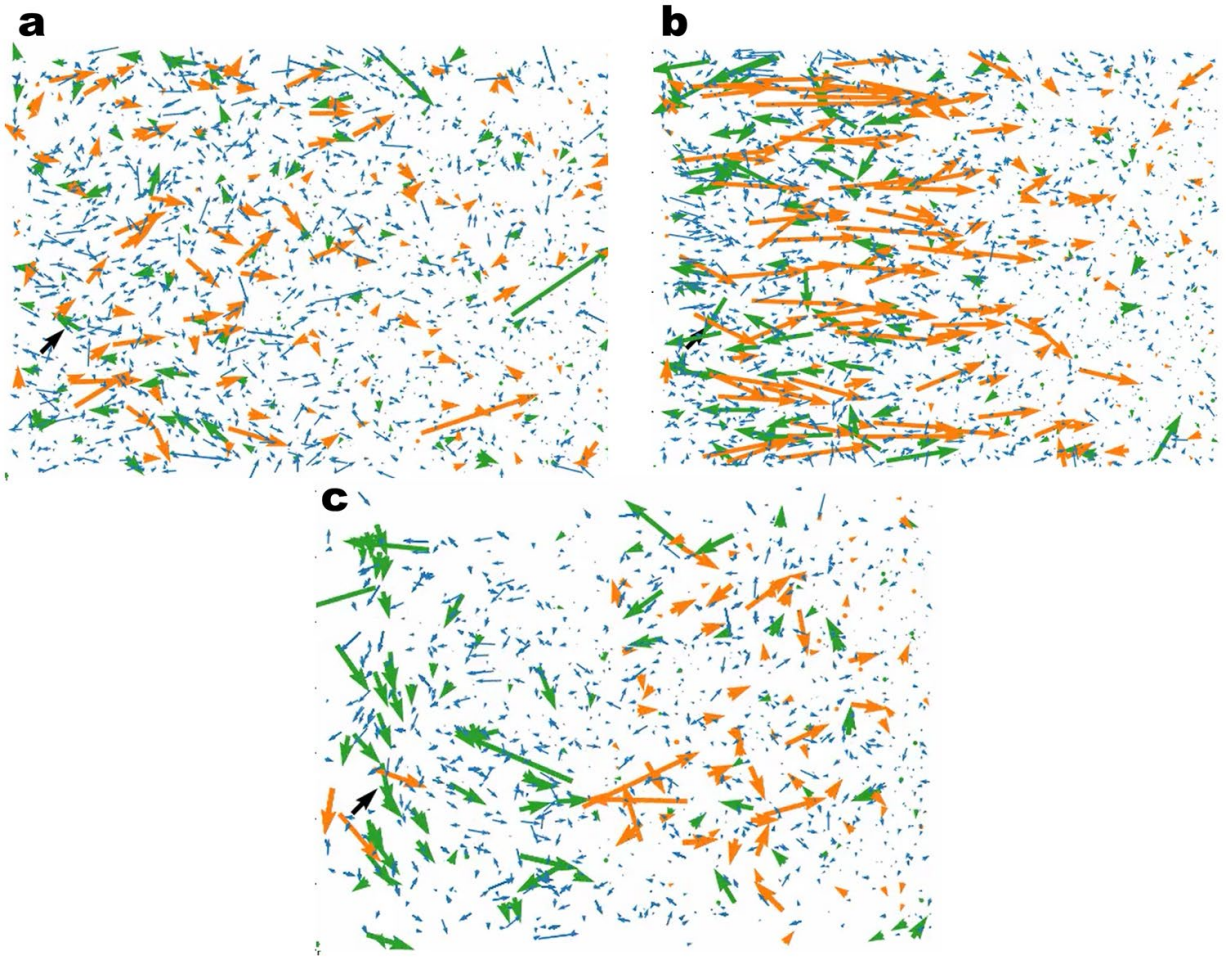
(**a**) Snapshot of the velocity field of the initial configuration. The small blue arrows indicate the EC solvent molecules, the green arrows indicate the Li$^{+}$ cations, and the orange arrows correspond to the PF6$^{-}$ anions. (**b**) Snapshot of the velocity field after the initiation of the first reactions in the reactive simulations. A tendency of orientation toward the electrode is seen for the Li$^{+}$ cations while the opposite for PF6$^{-}$. (**c**) A steady regime is reached where the ionic flux is removed.

**Figure 8:**
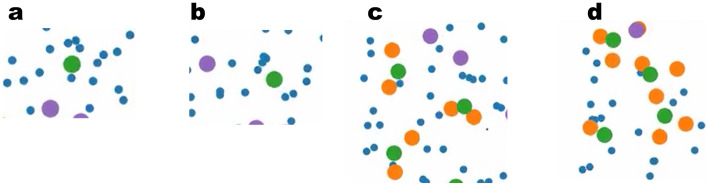
(**a**) An example snapshot of a coordination structure within the pre-reactive simulation. The green point indicates a Li$^{+}$ cation, the purple indicates PF6$^{-}$ anions, and the small blue points correspond to the double bonded oxygen atom of each EC molecule. Li$^{+}$ coordinating to $4{-}6$ EC molecules. (**b**) Same as (**a**). (**c**) A snapshot from reactive simulations after the initiation of the first reactions. The orange points indicate the closed radicals cEC$^{-}$. (LiEC$_{2}$)$^{-}$ clusters with the positive charge located in the middle between the two negative charges of the two radical anions. (**d**) Same as (**c**) after $\sim 100\; \text{ps}$. In addition one can recognize the formation of (LiEC$_{3}$)$^{2-}$ cluster with a trigonal geometry.

Having highlighted the properties of the graphite/carbonate model, we now turn to the decomposition kinetics. In Fig. 5a we show the total number of each radical and end-product molecular species as a function of time for $T\sim 10\; \; \text{ns}$. Starting from the initial configuration of the pure EC system, we can see that the products concentration is steeply increased within the first stage of $\sim 10^{-2}\; \text{ns}$ to reach a flat profile at longer times. The flattening of the profile after the initial stage is indicative of the onset of a regime where no valid reactive Li$^{+}$/EC clusters are found and the reactive clusters need to be reactivated through diffusive processes. The two conditions for the occurrence of the first reduction reaction consist of one EC located within a distance of $1\; \text{nm}$ from the electrode surface and one Li$^{+}$ coordinating to that EC. Since the number of EC molecules adsorbed at the electrode surface was only slightly changed due to the reactions, the rate limiting process for the first reduction corresponds to the coordination of Li$^{+}$ to EC and therefore also the flux of Li$^{+}$ from the bulk electrolyte to the reaction volume. We remark that for a diffusion constant of $D=5\times 10^{-5} \frac{{\text{nm}}^{2}}{\text{ps}}$ and a time window of $T=1 \; \; \text{ns}$ the displacement of Li$^{+}$ should be expected to be within the range of $1\; \; \text{nm}$, thus the diffusive flux of Li$^{+}$ into the reaction volume is not expected to contribute significantly to the reactivation of the EC reduction within the initial stage. Nevertheless, in Fig. 6a we show that the Li$^{+}$ number within the half unit cell containing the reaction volume shows a clear increase as a function of time after the initiation of the first reaction. We interpret this as a migration of Li$^{+}$ to the electrode driven by the excess of the negative charge transferred to the EC molecules in the first reaction. Figure 6b shows the time dependence of the electric charge within the reaction zone, indicating that the charge density within this region is initially decreased to negative values due to the reactions and then restored to charge-neutrality via the ionic flux. Thus, the transition from a high slope to a low slope in the turnover rate is characteristic of a transition from an activation limited to a diffusion or migration limited reduction kinetics. A further important observation is that the coordination of negatively charged cEC$^{-}$ radicals to Li$^{+}$ results in the formation of stable neutral and negatively charged (LiEC), (LiEC$_{2}$)$^{-}$ and (LiEC$_{3}$)$^{2-}$ bidentate and tridentate geometries (see Supplementary Fig. S4) prior to the onset of the ionic flux of Li$^{+}$ to the electrode. Since these complexes hinder the coordination of further EC molecules to Li$^{+}$, the formation of these stable geometries effectively results in a rate-limiting step prior to the onset of the Li$^{+}$ ionic flux to the electrode (see Figs. 7a–c and 8a–d).

**Figure 9:**
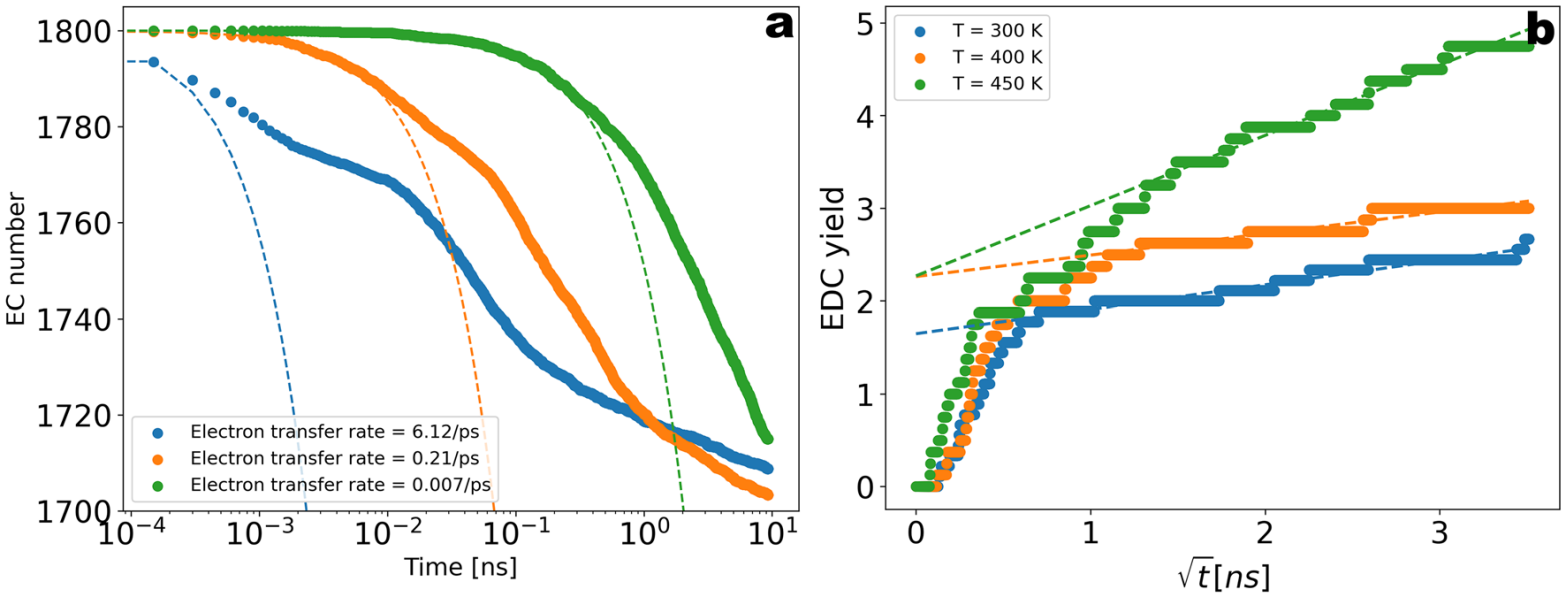
(**a**) Dependence on initial electron transfer rate. The activation energy is linearly varied between 0 to $16\; \text{kJ/mol}$. The time-dependence of the total number of EC molecules is shown, respectively. (**b**) Dependence on temperature. The plot shows faster degradation kinetics for higher temperatures, thus indicating thermally activated passivation film growth dynamics.

In order to explore the dependence of the observed growth kinetics on the charge transfer rate, the reactive simulations have been performed by varying the activation energy on a linear scale from 0 to 16 kJ/mol (see Supplementary Information), corresponding to rates $6.12\; \text{ps}^{-1}$, $2.1\times 10^{-1}\; \text{ps}^{-1}$, and $7\times 10^{-3}\; \text{ps}^{-1}$ in units of time (Fig. 9a). Remarkably, the observed reduction-induced flux of Li$^{+}$ ions to the electrode presented in Fig. 6a shows considerable sensitivity with respect to the charge transfer rate, thus underlining the crucial role of electro-neutrality for the observed effective reaction rates.

**Figure 10:**
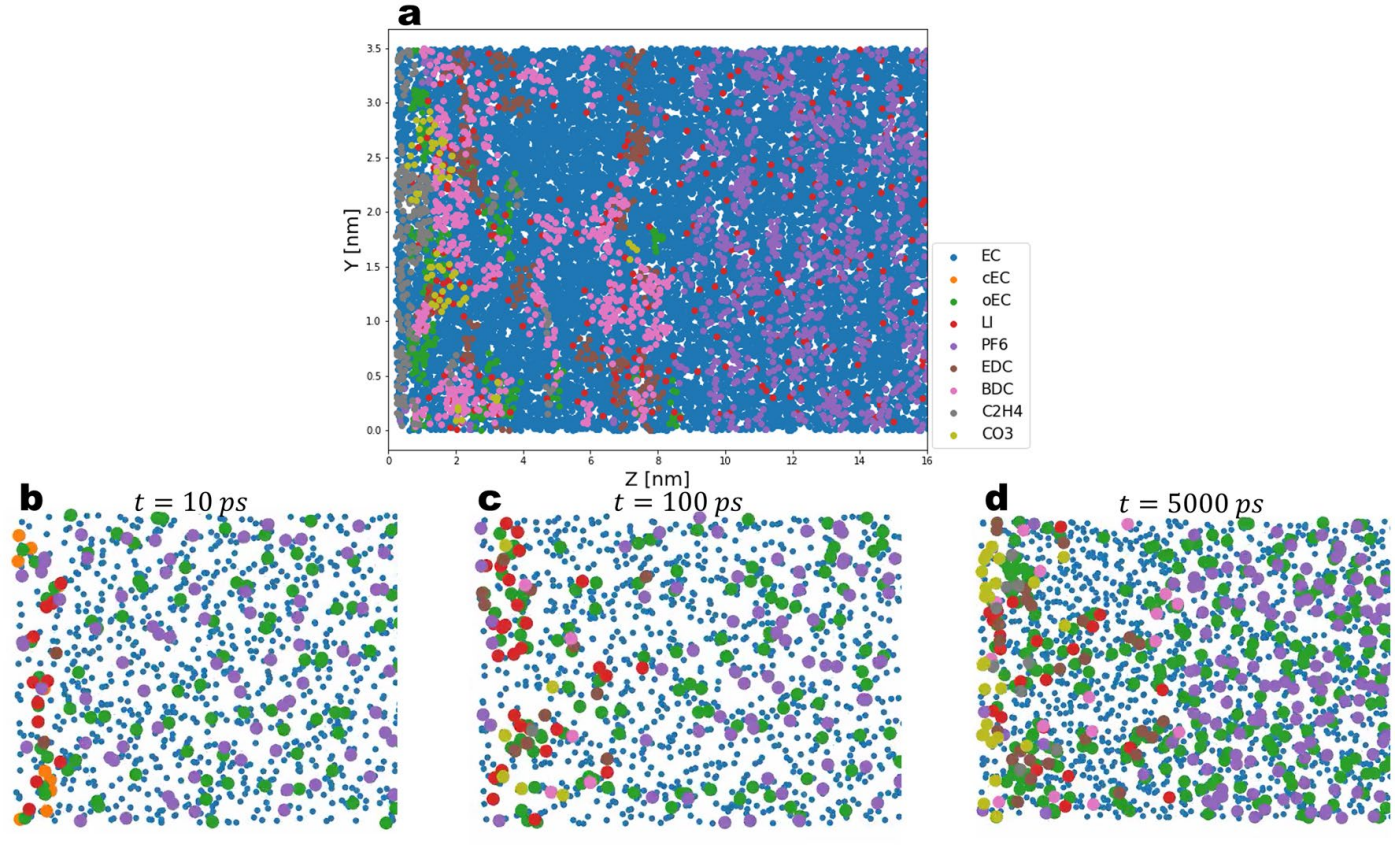
Side view of the slab at various stages of the SEI growth. The simplified representation shows each molecular species represented by one scatter point. (**a**) Complete simulation box at the end of the simulation, (**b**–**d**) Same as (**a**) at different times. Shown is the regime close to the electrode.

Continuing with the subsequent reactions, we observe that for the case of cEC$^{-}$ a tipping point is reached at an earlier stage due to the termination reactions where the oEC$^{-}$ is either fragmented to the doubly charged carbonate anion CO$_{3}^{2-}$ and C$_{2}$H$_{4}$ or a dimerization has taken place where two oEC$^{-}$ are condensed to yield BDC$^{2-}$ or EDC$^{2-}$ dimers. In Fig. 5b we show the *z*-density distribution of each molecular species. We can distinguish between two types of profiles; while the two dimer-like end-products show a broad peak around $z=7\; \text{nm}$ away from the electrode, lithium carbonate has sharper density distribution with a peak located closer to the electrode. The broad profile of the dimer species is due to the diffusion and migration of the radical cEC$^{-}$ away from the electrode, implying that the dimerization reactions do not only occur near the electrode. On the other hand, the sharper density of lithium carbonate could be explained by its relatively small solubility. Overall the qualitative aspects of the formed layer are in line with the general understanding of a dense inorganic layer at the electrode side and organic compounds at the electrolyte side (see Fig. 10a–d).

The temperature dependence of the time-evolution of the Li$_{2}$EDC is shown in Fig. 9b. Faster kinetics are seen for higher temperatures, which is consistent with the Arrhenius definition of the elementary rates. In Fig. 9b we can again distinguish between two different time windows where the growth rate shows two different variations as a function of temperature. These two variations are in qualitative agreement with the previous finding of the transition from a reaction-limited growth kinetics to a diffusion limited regime. In Fig. 9b the time axis was scaled as the square root of time to indicate the transition to the diffusion-limited regime suggested by the linear relation Li$_{2}$EDC $yield\sim \, \sqrt{t}$. From the yield rates ${\dot{n}}_{EDC}$ for the two temperatures $T=300\; \; \text{K}$ and $T=450 \; \; \text{K}$ the activation energy is estimated as $E \sim 3\; \text{kJ/mol}$. We note that the temperature dependence of capacity fading rates reported in experimental works based on cycling experiments is often identified with the temperature dependence of the growth rate of the SEI (see Supplementary Table S4 and references therein). In the indicated references an activation energy within the range of $30{-}50\; \text{kJ/mol}$ was identified based on the slope of the logarithmic rate of capacity loss versus the inverse temperature. Although making quantitative comparisons with these results goes beyond our scope due to the short simulation-time and the possibly related transient effects, it is in principle possible to explore the $\upmu s$ range within our framework and this could be therefore done in future work, thus providing a mechanistic framework for investigating thermally activated SEI growth processes. Overall, the results of our simulations suggest the onset of a Li$^{+}$ flux to the electrode as well as the formation of (LiEC$_{2}$)$^{-}$ and (LiEC$_{3}$)$^{2-}$ complexes as rate limiting factors within the *ns* time-scale.

**Figure 11:**
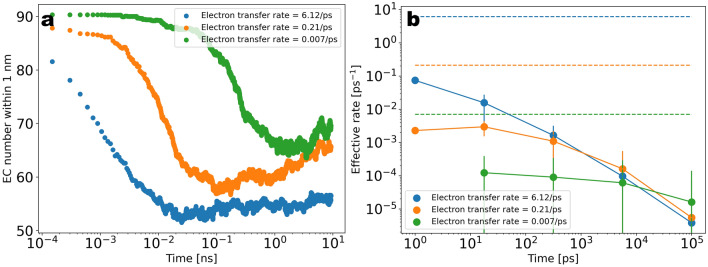
(**a**) Number of EC molecules in analogy to Fig. 9a but restricted ot a range within $1\; \text{nm}$ from the electrode (the reaction cell). (**b**) Comparison of the effective reaction rate with the initial electron transfer rate. The effective rate is calculated from the slope of EC number vs time normalized by the EC number within the reaction cell. To reduce the fluctuations the time-axis is logarithmically binned. The negative effective rate $-\frac{\dot{n_{EC}}}{n_{EC}}$ is shown for a logarithmic $y$-axis.

To explore the kinetics somewhat more quantitatively, we specifically analyse the number of EC molecules in the reaction cell, defined by the distance of at most $1\; \text{nm}$ from the electrode. Again the elementary electron transfer rate has been systematically varied. As seen in Fig. 11a, the reduction rate is effectively reduced to considerably smaller values at longer times due to the observed diffusion and migration effects. For a quantitative comparison of the effective rates, we have calculated the normalized rate $\nu _{eff} = \frac{\dot{n_{EC}}}{n_{EC}}$ by extracting the numerical time-derivative from the EC molecular number from Fig. 9a normalized by the EC number within the reaction cell shown in Fig. 11a. The result is shown in Fig. 11b. For short times the effective rates are proportional to the elementary rates (dashed lines). The difference expresses the necessity of the presence of a nearby lithium ion to perform a reaction. After the initial transient regime a transition to the “diffusion-controlled regime” can be observed, where the elementary electron transfer rate has little influence and the effective reaction rate is limited by the available number of Li$^{+}$ ions.

### Post-reactive simulations

**Figure 12:**
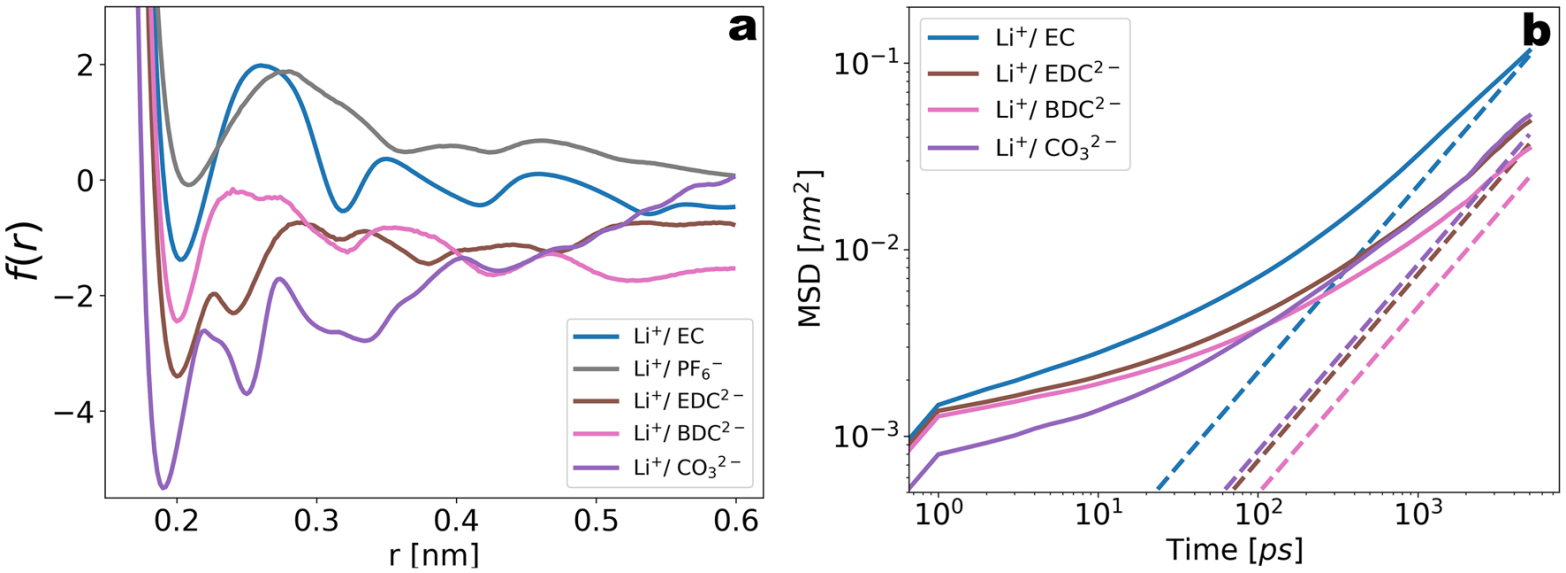
(**a**) Potential of mean force (PMF) based on the logarithmic radial distribution functions (RDF) of Li$^{+}$ with the reaction products CO$_{3}^{2-}$, BDC$^{2-}$, EDC$^{2-}$, cEC$^{-}$ and oEC$^{-}$. Compared to the Li$^{+}$/EC PMF, the depth of the first minimum is increased by a factor of $5{-}7$, reflecting a stronger binding affinity. (**b**) MSD vs time for Li$^{+}$ coordinated the product species. The MSD labeled with Li$^{+}$/EC coordination was extracted from pre-reactive simulations of the baseline system (see also Fig. 4), while the remaining MSDs were extracted from post-reactive MD runs.

In the previous section the emphasis was on the growth dynamics of the passivation layer and the underlying mechanisms. We now consider the structures generated from the reactive simulations and focus on how they compare to the baseline electrolyte. Previous work addressing this question relied on manual generation of amorphous or crystalline structures of known passivation film components, such as Li$_{2}$BDC and Li$_{2}$EDC, and attempted to infer conductivity or activation energies from these structures^54–56^. Remarkably, the authors showed that the diffusivity and activation energy of Li$^{+}$ within Li$_{2}$EDC compares well with experimental measurements of the conductivity of synthetic SEI compounds^54^. Although this has allowed a precise interpretation of the conduction properties of passivation layers, a drawback of that procedure is the manual generation of the amorphous or crystalline structures of the passivation film components. An important aspect of our work is to allow deriving these structures based on the initial liquid electrolyte structure. In order to illustrate this in more detail, we show the potential of mean force (PMF) based on the RDFs of Li$^{+}$ with the decomposition products during the reactive simulation in Fig. 12a. A remarkable distinction with respect to the Li$^{+}$/EC PMF is that the depth of the first minimum is considerably increased. We suggest that this increase in the barrier depth is caused by the stabilizing ionic forces between the Li$^{+}$ and the negatively charged degradation products. In order to explore the effect of this increased binding affinity on the dynamic properties, we show in Fig. 12b the time-dependence of the Li$^{+}$ MSD labeled with the corresponding coordination environment. It can be seen that EDC$^{2-}$ and BDC$^{2-}$ coordinated Li$^{+}$s show a slower diffusion compared to Li$^{+}$/EC structures. Importantly, the labeled structures (Li$^{+}$ coordinated to CO$_{3}^{2-}$, EDC$^{2-}$ and BDC$^{2-}$) were observed to be stable within the time window used for calculating the MSD (Fig. S7 in Supplementary Information), thus implying a well-defined structure-dynamics correlation. Overall the observations qualitatively agree with the result that the SEI-activation energy is considerably larger than the activation of the carbonate liquid electrolyte (see Supplementary Table S4).

## Discussion

We have investigated SEI-formation reactions within a time-scale of few nanoseconds based on our rs@md simulation of the graphite/carbonate electrolyte model. Prior to the reactions the structural and dynamic properties of the model were consistently validated in terms of the baseline density and the diffusivity values. We have suggested the formation of negatively charged Li$^{+}$/EC$^{-}$ structures as well as the flux of Li$^{+}$s from the bulk electrolyte to the electrode as rate limiting steps for the EC reduction reaction. The effect of the Li$^{+}$ flux or “concentration polarization” was observed at high reaction rates. Since concentration polarization occurs in general at much longer times, this could be a consequence of the relatively large electron transfer rates used in our simulation. Nevertheless, our results give insights into the role of electro-neutrality and the structure of the EDL in determining the effective rates. Our results were based on the Li$^{+}$ injection scheme accounting for the charge transfer reactions at the interface to maintain electro-neutrality. Importantly, the surface charge at the graphite anode was assumed to be fixed in our simulations and a treatment accounting for charge fluctuations at the electrode surface due to the reactions could be considered in future work to account for the proper voltage dependence of the decomposition process^57–59^. As the elementary electron-transfer rates were chosen based on the accessible time-range of our simulation, the derivation of these elementary rates from more rigorous frameworks (i.e Marcus theory) would be viewed as complementary to our work. Overall, our work shows how gas-phase energy barriers can be used to derive liquid-phase reaction rates based on the rs@md framework. The implications of the changed chemical environments on Li$^{+}$ diffusivity and coordination was also discussed. Next steps could be the consideration of more complex or more realistic electrolyte formulations, with the attempt of identifying the functionality of known additive compounds and their effect on the calculated growth rates. Using the derived structures to calculate observable mechanical properties (i.e bulk modulus) could also be of interest.

## Supplementary Information

Supplementary Information.

## Data Availability

Source code and data can be obtained from the corresponding author upon request.

## References

[1] Winter, M. & Brodd, R. J. What are batteries, fuel cells, and supercapacitors?. *Chem. Rev.* **104**, 4245–4270 (2004). 15669155 10.1021/cr020730k

[2] Winter, M., Barnett, B. & Xu, K. Before Li ion batteries. *Chem. Rev.* **118**, 11433–11456 (2018). 30500179 10.1021/acs.chemrev.8b00422

[3] Xu, K. Electrolytes and interphases in Li-ion batteries and beyond. *Chem. Rev.* **114**, 11503–11618 (2014). 25351820 10.1021/cr500003w

[4] Xu, K. Nonaqueous liquid electrolytes for lithium-based rechargeable batteries. *Chem. Rev.* **104**, 4303–4418 (2004). 15669157 10.1021/cr030203g

[5] Weiling, M., Pfeiffer, F. & Baghernejad, M. Vibrational spectroscopy insight into the electrode|electrolyte interface/interphase in lithium batteries. *Adv. Energy Mater.* **12**, 2202504 (2022).

[6] Adenusi, H., Chass, G. A., Passerini, S., Tian, K. V. & Chen, G. Lithium batteries and the solid electrolyte interphase (SEI)-progress and outlook. *Adv. Energy Mater.* **13**, 2203307 (2023).

[7] Diddens, D. *et al.* Modeling the solid electrolyte interphase: Machine learning as a game changer?. *Adv. Mater. Interfaces* **9**, 2101734 (2022).

[8] Horstmann, B., Single, F. & Latz, A. Review on multi-scale models of solid-electrolyte interphase formation. *Curr. Opin. Electrochem. Fundam. Theor. Electrochem.-Phys. Nanoelectrochemistry* **13**, 61–69 (2019).

[9] Gross, A. Ab initio molecular dynamics simulations of reactions at surfaces. *Phys. Status Solidi (b)* **217**, 389–404 (2000).

[10] Chandrasekhar, J., Smith, S. F. & Jorgensen, W. L. Theoretical examination of the SN2 reaction involving chloride ion and methyl chloride in the gas phase and aqueous solution. *J. Am. Chem. Soc.* **107**, 154–163 (1985).

[11] Chandrasekhar, J., Smith, S. F. & Jorgensen, W. L. SN2 reaction profiles in the gas phase and aqueous solution. *J. Am. Chem. Soc.* **106**, 3049–3050 (1984).

[12] Takenaka, N., Suzuki, Y., Sakai, H. & Nagaoka, M. On electrolyte-dependent formation of solid electrolyte interphase film in lithium-ion batteries: Strong sensitivity to small structural difference of electrolyte molecules. *J. Phys. Chem. C* **118**, 10874–10882 (2014).

[13] Abbott, J. W. & Hanke, F. Kinetically corrected Monte Carlo-molecular dynamics simulations of solid electrolyte interphase growth. *J. Chem. Theory Comput.* **18**, 925–934 (2022). 35007421 10.1021/acs.jctc.1c00921

[14] Biedermann, M., Diddens, D. & Heuer, A. Connecting the quantum and classical mechanics simulation world: Applications of reactive step molecular dynamics simulations. *J. Chem. Phys.* **154**, 194105 (2021). 34240915 10.1063/5.0048618

[15] Biedermann, M., Diddens, D. & Heuer, A. rs@md: Introducing reactive steps at the molecular dynamics simulation level. *J. Chem. Theory Comput.* **17**, 1074–1085 (2021). 33497226 10.1021/acs.jctc.0c01189

[16] Ploehn, H. J., Ramadass, P. & White, R. E. Solvent diffusion model for aging of lithium-ion battery cells. *J. Electrochem. Soc.* **151**, A456 (2004).

[17] Pinson, M. B. & Bazant, M. Z. Theory of SEI formation in rechargeable batteries: Capacity fade, accelerated aging and lifetime prediction. *J. Electrochem. Soc.* **160**, A243 (2012).

[18] Li, D. *et al.* Modeling the SEI-formation on graphite electrodes in LiFePO_4_ batteries. *J. Electrochem. Soc.* **162**, A858 (2015).

[19] Winter, M., Novák, P. & Monnier, A. Graphites for lithium-ion cells: The correlation of the first-cycle charge loss with the Brunauer–Emmett–Teller surface area. *J. Electrochem. Soc.* **145**, 428 (1998).

[20] Joho, F. *et al.* Relation between surface properties, pore structure and first-cycle charge loss of graphite as negative electrode in lithium-ion batteries. *J. Power Sources* **97–98**, 78–82 (2001).

[21] Kasnatscheew, J. *et al.* A tutorial into practical capacity and mass balancing of lithium ion batteries. *J. Electrochem. Soc.* **164**, A2479 (2017).

[22] Lin, Y.-X. *et al.* Connecting the irreversible capacity loss in Li-ion batteries with the electronic insulating properties of solid electrolyte interphase (SEI) components. *J. Power Sources* **309**, 221–230 (2016).

[23] Kohs, W. *et al.* A study on electrolyte interactions with graphite anodes exhibiting structures with various amounts of rhombohedral phase. *J. Power Sources* **119–121**, 528–537 (2003).

[24] Vogl, U. S. *et al.* The mechanism of SEI formation on single crystal Si(100), Si(110) and Si(111) electrodes. *J. Electrochem. Soc.* **162**, A2281 (2015).

[25] Li, T. & Balbuena, P. B. Theoretical studies of the reduction of ethylene carbonate. *Chem. Phys. Lett.* **317**, 421–429 (2000).

[26] Jorgensen, W. L., Maxwell, D. S. & Tirado-Rives, J. Development and testing of the OPLS all-atom force field on conformational energetics and properties of organic liquids. *J. Am. Chem. Soc.* **118**, 11225–11236 (1996).

[27] Canongia Lopes, J. N., Deschamps, J. & Pádua, A. A. H. Modeling ionic liquids using a systematic all-atom force field. *J. Phys. Chem. B* **108**, 2038–2047 (2004).

[28] Abraham, M. J. *et al.* GROMACS: High performance molecular simulations through multi-level parallelism from laptops to supercomputers. *SoftwareX* **1–2**, 19–25 (2015).

[29] Martínez, L., Andrade, R., Birgin, E. G. & Martínez, J. M. PACKMOL: A package for building initial configurations for molecular dynamics simulations. *J. Comput. Chem.* **30**, 2157–2164 (2009). 19229944 10.1002/jcc.21224

[30] Reinier, L. C., Akkermans, N. A. S. & Robertson, S. H. COMPASS III: Automated fitting workflows and extension to ionic liquids. *Mol. Simul.* **47**, 540–551 (2021).

[31] , M. S. Dassault Systémes (2022), Vélizy-Villacoublay, France (2023).

[32] Nie, M. *et al.* Effect of vinylene carbonate and fluoroethylene carbonate on SEI formation on graphitic anodes in Li-ion batteries. *J. Electrochem. Soc.* **162**, A7008 (2015).

[33] Naji, A., Ghanbaja, J., Humbert, B., Willmann, P. & Billaud, D. Electroreduction of graphite in LiClO_4_-ethylene carbonate electrolyte. Characterization of the passivating layer by transmission electron microscopy and Fourier-transform infrared spectroscopy. *J. Power Sources* **63**, 33–39 (1996).

[34] Kuai, D. & Balbuena, P. B. Solvent degradation and polymerization in the Li-metal battery: Organic-phase formation in solid-electrolyte interphases. *ACS Appl. Mater. Interfaces* **14**, 2817–2824 (2022). 34994191 10.1021/acsami.1c20487

[35] Leung, K. & Budzien, J. L. Ab initio molecular dynamics simulations of the initial stages of solid-electrolyte interphase formation on lithium ion battery graphitic anodes. *Phys. Chem. Chem. Phys.* **12**, 6583–6586 (2010). 20502786 10.1039/b925853a

[36] Vogl, U. S. *et al.* The mechanism of SEI formation on a single crystal Si(100) electrode. *J. Electrochem. Soc.* **162**, A603 (2015).

[37] Wang, Z., Yang, Y., Olmsted, D. L., Asta, M. & Laird, B. B. Evaluation of the constant potential method in simulating electric double-layer capacitors. *J. Chem. Phys.* **141**, 184102 (2014). 25399127 10.1063/1.4899176

[38] Scalfi, L., Salanne, M. & Rotenberg, B. Molecular simulation of electrode-solution interfaces. *Annu. Rev. Phys. Chem.* **72**, 189–212 (2021). 33395545 10.1146/annurev-physchem-090519-024042

[39] Zhang, C., Sayer, T., Hutter, J. & Sprik, M. Modelling electrochemical systems with finite field molecular dynamics. *J. Phys. Energy* **2**, 032005 (2020).

[40] Morita, M., Asai, Y., Yoshimoto, N. & Ishikawa, M. A Raman spectroscopic study of organic electrolyte solutions based on binary solvent systems of ethylene carbonate with low viscosity solvents which dissolve different lithium salts. J. Chem. Soc. *Faraday Trans.* **94**, 3451–3456 (1998).

[41] Xu, K. “Charge-Transfer’’ process at graphite/electrolyte interface and the solvation sheath structure of Li+ in nonaqueous electrolytes. *J. Electrochem. Soc.* **154**, A162 (2007).

[42] Kempa, R. & Lee, W. H. 392. The dipole moments of some cyclic carbonates. *J. Chem. Soc.* 1936–1938 (1958).

[43] Vatamanu, J., Borodin, O. & Smith, G. D. Molecular dynamics simulation studies of the structure of a mixed carbonate/LiPF_6_ electrolyte near graphite surface as a function of electrode potential. *J. Phys. Chem. C* **116**, 1114–1121 (2012).

[44] Mozhzhukhina, N. *et al.* Direct operando observation of double layer charging and early solid electrolyte interphase formation in Li-ion battery electrolytes. *J. Phys. Chem. Lett.* **11**, 4119–4123 (2020). 32354215 10.1021/acs.jpclett.0c01089 PMC7467741

[45] Benjamin, I. Chemical reactions and solvation at liquid interfaces: A microscopic perspective. *Chem. Rev.* **96**, 1449–1476 (1996). 11848798 10.1021/cr950230+

[46] Becker, M. *et al.* Multiscale modeling of aqueous electric double layers. *Chem. Rev.* **124**, 1–26 (2024). 38118062 10.1021/acs.chemrev.3c00307 PMC10785765

[47] Schmickler, W. Electronic effects in the electric double layer. *Chem. Rev.* **96**, 3177–3200 (1996). 11848857 10.1021/cr940408c

[48] Wu, J. Understanding the electric double-layer structure, capacitance, and charging dynamics. *Chem. Rev.* **122**, 10821–10859 (2022). 35594506 10.1021/acs.chemrev.2c00097

[49] Jeanmairet, G., Rotenberg, B. & Salanne, M. Microscopic simulations of electrochemical double-layer capacitors. *Chem. Rev.* **122**, 10860–10898 (2022). 35389636 10.1021/acs.chemrev.1c00925 PMC9227719

[50] Wu, Q., McDowell, M. T. & Qi, Y. Effect of the electric double layer (EDL) in multicomponent electrolyte reduction and solid electrolyte interphase (SEI) formation in lithium batteries. *J. Am. Chem. Soc.* **145**, 2473–2484 (2023). 36689617 10.1021/jacs.2c11807 PMC9896563

[51] Solchenbach, S., Huang, X., Pritzl, D., Landesfeind, J. & Gasteiger, H. A. Monitoring SEI formation on graphite electrodes in lithium-ion cells by impedance spectroscopy. *J. Electrochem. Soc.* **168**, 110503 (2021).

[52] Thum, A., Diddens, D. & Heuer, A. Impact of charged surfaces on the structure and dynamics of polymer electrolytes: Insights from atomistic simulations. *J. Phys. Chem. C* **125**, 25392–25403 (2021).

[53] Hayamizu, K. Temperature dependence of self-diffusion coefficients of ions and solvents in ethylene carbonate, propylene carbonate, and diethyl carbonate single solutions and ethylene carbonate + diethyl carbonate binary solutions of LiPF6 studied by NMR. *J. Chem. Eng. Data* **57**, 2012–2017 (2012).

[54] Borodin, O., Zhuang, G. V., Ross, P. N. & Xu, K. Molecular dynamics simulations and experimental study of lithium ion transport in dilithium ethylene dicarbonate. *J. Phys. Chem. C* **117**, 7433–7444 (2013).

[55] Muralidharan, A., Chaudhari, M., Pratt, L. & Rempe, S. Molecular dynamics of lithium ion transport in a model solid electrolyte interphase. *Sci. Rep.* **8**, 10736 (2018). 30013026 10.1038/s41598-018-28869-x PMC6048109

[56] Benitez, L. & Seminario, J. M. Ion diffusivity through the solid electrolyte interphase in lithium-ion batteries. *J. Electrochem. Soc.* **164**, E3159 (2017).

[57] Leung, K. & Tenney, C. M. Toward first principles prediction of voltage dependences of electrolyte/electrolyte interfacial processes in lithium ion batteries. *J. Phys. Chem. C* **117**, 24224–24235 (2013).

[58] Li, Y. & Qi, Y. Energy landscape of the charge transfer reaction at the complex Li/SEI/electrolyte interface. *Energy Environ. Sci.* **12**, 1286–1295 (2019).

[59] Leung, K. Predicting the voltage dependence of interfacial electrochemical processes at lithium-intercalated graphite edge planes. *Phys. Chem. Chem. Phys.* **17**, 1637–1643 (2015). 25438093 10.1039/c4cp04494k

