# Supplementary material for: Reactive molecular dynamics simulations of lithium-ion battery electrolyte degradation
Source: Sci Rep. 2024 May 4;14:10281. doi: 10.1038/s41598-024-60063-0 (PMC11584793; doi:10.1038/s41598-024-60063-0)
Supplement: Supplementary file 1 — Supplementary Information. [file 41598_2024_60063_MOESM1_ESM.pdf]

Supplementary Information for Reactive molecular dynamics simulations of Lithium-ion battery electrolyte degradation

Youssef Mabrouk,† Nehzat Safaei,‡ Felix Hanke,*,¶ Johan M. Carlsson,¶ Diddo Diddens,† and Andreas Heuer*,§

†*Forschungszentrum Jülich GmbH, Helmholtz-Institute Münster (IEK-12), Corrensstraße 46, 48149 Münster, Germany*

‡*Dassault Systèmes Deutschland GmbH, Am Kabellager 11-13, D-51063 Cologne, Germany*

¶*Dassault Systèmes, Cambridge CB4 0WN, U.K.*

§*Institute of Physical Chemistry, University of Münster, Corrensstrasse 28/30, 48149 Münster, Germany*

E-mail: felix.hanke@3ds.com; andheuer@wwu.de

Reaction rates and further parameters

The reaction probability for a given coordinated complex is defined according to transition state theory via the gas-phase activation energy of the corresponding reaction. The reaction barriers used in this work for ring-opening and polymerization reactions are based on the values reported in our previous work [1]. For electron transfer reactions a more extensive literature query was performed. The values extracted from literature were summarized in Table S1, with the basic system descriptions and the correspondingly used simulation method. Generally, investigations based on AIMD simulations report reaction rates within

the range of 10^{-2} to $10^{0}\, ps^{-1}$, which is just the time range of AIMD simulations. More efficient simulation frameworks like MDMC or ReaxFF methods imply smaller rates within the ps^{-1} to ns^{-1} range, which is also the rough time-scale of MD simulations. We attribute this considerable difference between the reported values to the time limits of the mentioned simulation methods. We note that the reaction rates reported in the mentioned works can not be directly compared with experimental reaction rates, since typically reported values of the exchange current density lie within the 10^{-1} to $10^{1}\, mA/cm^2$, which would effectively correspond to a rate μs^{-1} in units of time if a reaction region of $1\, nm$ and concentration of $1\, mol/L$ is assumed. We conclude that the available published data on EC reduction do not allow the identification of a well-defined reaction rate on an absolute time scale. Nevertheless these studies clearly provide considerable insight into the reduction mechanisms within the simulated time scale. Our strategy for the choice of the input reduction rate was to perform a scan of all possible electron transfer rates that can be accessed within our simulation time. This was performed by defining a linear scale of activation energy, going from 0 to 16 kJ/mol, and calculating the rate based on the law $\nu = \frac{k_B T}{h} \times exp(-\frac{E_a}{k_B T})$. Thus, assuming a barrier-less reaction the rate would correspond to $6.12\, ps^{-1}$, and each increment in the activation energy yields a decay with roughly one order of magnitude in units of time. This strategy allows us to gain insight into the mechanisms can possibly occur at the force field level (from ps to μs) independently from the reliability of the input information. Nevertheless we emphasize that the goal of this framework is to allow the use of reaction barriers and related information from first principles calculations. Thus, single point calculations at the TPSS level of theory [2] in the 6-31G set [3] were performed using the Gaussian software[4] in order to investigate the barrier for the reduction reaction. This was done by optimizing one single positively charged $LiEC_6^+$ solvation structure extracted from the MD data and calculating the vertical and adiabatic energy differences subsequent to an addition of an electron. The solvent polarization beyond the nearest neighbour hydration shell was considered by using polarizable continuum model (PCM) for the single point calculations [5]

with the permittivity of water. A comparison of the calculated energy difference between EC and cEC^- or ($LiEC_6^+$ and $LiEC_6$) shows that the adiabatic difference is in agreement with the reduction potential value of $0.6 - 1.0\,V$ vs Li/Li^+. In Table S6 we have summarized the experimental values of the reduction potential of EC and in Figure S2 the results of the DFT-barriers are summarized. After the first step of electron addition and the second step of geometry optimization, a second electron was added and the geometry optimization was repeated in order to estimate the effect of an cEC^- radical coordinating within the cluster on the reduction barrier. These results were verified using DMol3 [6] with the same geometries. The computations were done using TPSS functional + COSMO implicit solvent with relative dielectric constant 95.3. Notably, the first energy difference is within $0.03\,eV$ of discrepancy ($0.75\,eV$ vs $0.71\,eV$), while the subsequent electron addition steps show larger differences ($\sim 0.5\,eV$). This is to be expected due to the excess of negative charge imposed on the system. In order to extract the reduction barrier from the vertical and adiabatic energies, and quantify how this barrier depends on the presence of cEC^- radicals within the solvation shell, we have used the Marcus transfer rate $e^{\frac{-(\delta G+\lambda)^2}{4\lambda k_B T}}$ identifying δG as the difference between the initial state and the geometry-optimized reduced state, while λ is identified as the difference between the reduced state and the geometry optimized reduced state. In Figure S2 this is equivalent to $\delta G = E_0 - E_2$ and $\lambda = E_1 - E_2$ for the first reduction. Thus the barrier of the first reduction is $\delta G_1^* = 7.39$ while the barrier for the second reduction is $\delta G_2^* = 13.3$.

A further important aspect that needs to be considered is the effect of the coordinating cEC^- radical on the electron tunneling rate compared to the cluster without cEC^-. Indeed, if the tunneling from the electrode to the EC molecule is considered as a simplified tunneling process between two structure-less potential wells, then the consideration of the coordinated cEC^- corresponds to adding a repulsive potential near the potential well representing the EC molecule. This would clearly lead to an increase to the barrier between the two potential wells. In order to implement a simplified minimal model for estimating this effect, we have

calculated the tunneling matrix element between a Li atom and neutral EC molecule, and repeated the calculation with adding one cEC^- radical near the EC. The Lithium atom is used as a minimal model for the electron source corresponding to the electrode, or the first potential well, and the second potential well corresponds to the EC molecule and represents the electron sink. In order to compute the tunneling rate from the Lithium atom (the fictive electrode or the source) to the EC molecule (the sink), we first perform unconstrained Hartree Fock (UHF) calculations in the 6-31+G* atomic orbital (AO) basis of the Lithium atom and the EC molecule separately to generate the corresponding AO vectors. The obtained AO vectors are then used as initial conditions for a single point calculation based on the combined geometries to obtain an AO vector of the Li/EC structure with the electron localised on the Li atom. Once this is done, the calculation is repeated for a Li^+ cation and a negatively charged cEC^- molecule leading to Li^+/EC- configuration where the electron is localised on the EC molecule. The tunneling matrix element is then calculated based on these configurations using the electron transfer (ET) module [7] of NWChem software package [8]. Finally the procedure is repeated with an added cEC^- anion near the EC molecule. As expected, a decrease by a factor of 10^{-2} in the tunneling rate was observed upon adding the cEC^- to the coordination structure due to the increase of the tunneling barrier via the Coulomb repulsion between the cEC^- and the added electron. However we emphasize that this estimation does not take several details such as the electrode structure and the electrolyte structure into account, and is therefore only a crude approximation of the change in the tunneling rate due to coordinating cEC^- radicals.

Charge neutralization

An important technical aspect considered in this work is the compensation of the electric charge in the simulation slab due to the charge transfer reactions. In principle, the method used for the neutralisation of electric charge depends on the studied system or situation. For example, simulating the dissolution of a Lithium metal electrode into Lithium ions

and negatively charged solvent molecules would not require specific charge neutralization schemes, since the simulation cell would stay overall neutral. But for electron transfer reactions from the electrode to the electrolyte not involving dissolution of Li^+ ions to the electrolyte, it is necessary to compensate each added negative charge for each electron with a positive charge. The neutralisation method used in this work consists of inserting a Li^+ cation into the simulation cell at the opposite side of the reaction region for each reduction reaction. In a realistic system we expect the negative charge resulting from the reactions at the interface to be compensated by a Li^+ flux from the bulk electrolyte, as well as a flux of anions toward the bulk electrolyte. Inserting Li^+ cations in the bulk region allows therefore to simulate this ionic flux. Concretely, this is implemented by inserting *initially* frozen LJ-particles to the region within $[L_{z/2}, L_z]$ of the slab, while the reaction region is confined within $[0, 1\,nm]$ from the slab, thus sufficiently far from the insertion region. With each reduction reaction, *the freezing of one LJ-particle is removed* and the LJ particle is turned to a Li^+ cation. Adding the LJ-particles to the initial configuration instead of adding them during the reactive steps allows to avoid instabilities occurring due overlapping volumes. We emphasise that the motivation of our approach is to allow a consistent implementation of the Li^+ flux from the bulk electrolyte to the electrode. Adding the Li^+s randomly across the slab could imply a local increase of the Li^+ concentration near the electrode which is not physical, thus effectively biasing the reaction rates. On the other hand, in a realistic electrochemical environment the excess charge on the electrolyte should be compensated by an ionic flux from the bulk electrolyte.

Density variations

A further important aspect consists of the variation of the density during the reactive simulations. The reference density of the non-reactive simulated system corresponds to 1.38 g/cm3. However the density was expected to show variations due to the reactions, since for example adding ionic forces to the system can locally increase the density, or the addition and

removal of bonded forces could similarly also contribute to density variations. The change of the density during the reactive simulations was therefore monitored, and it was concluded that the density change is within 1 percent of the reference value. This small variation is explained by the overall relatively small number of reaction performed and suggests that longer simulation times could be needed to observe a more pronounced aggregation of the product molecules. The effect of inserting Li+ ions to the unit cell was also shown to result in density variations within one percent of the total density and was therefore not considered further.

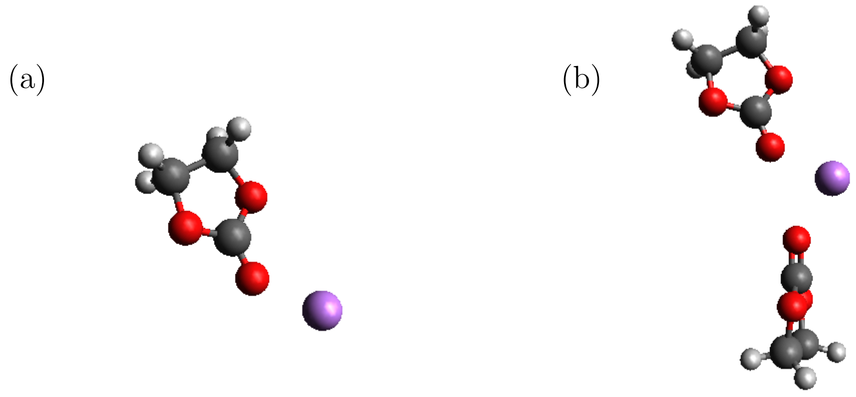

Supplementary Figure S 1: Simplified model for the estimation of the cEC^- effect on the tunneling rate. In (a) the tunneling matrix element between Li/EC and Li^+/EC^- configurations is calculated. In (b) the same calculation is repeated with adding a negatively charged cEC^- molecule to the structure.

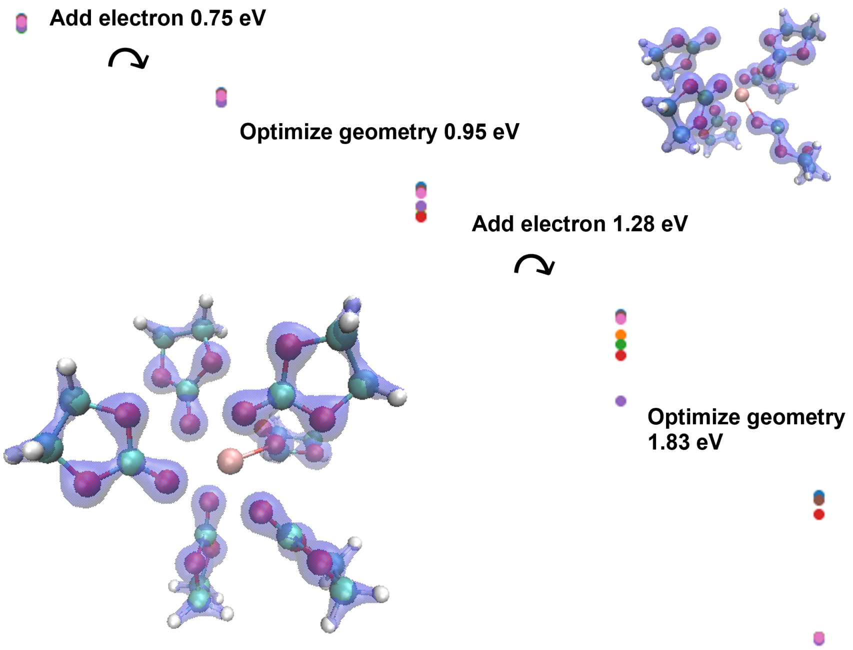

Supplementary Figure S 2: Simplified model used for the estimation of the reorganization energy and the effect of the solvation shell on the reorganization energy. The first step with an energy drop of $\delta E = 0.75$ eV corresponds to addition of one electron to $(LiEC_6)^+$. The second step with $\delta E = 0.95$ eV corresponds to geometry optimization of $LiEC_6$. The third and fourth steps corresponds again to electron addition and geometry optimization of the $LiEC_6$ complex. The different points indicate multiple initial geometries.

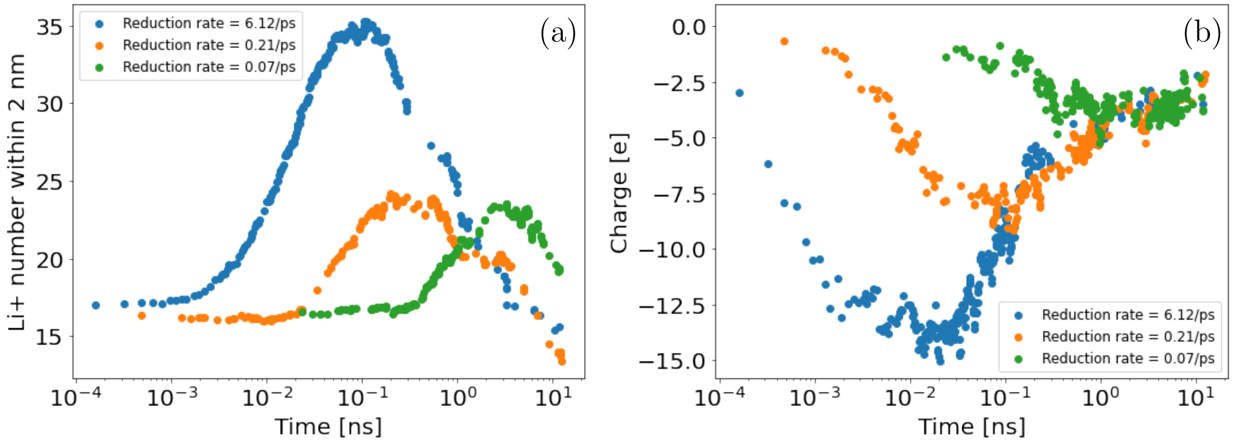

Supplementary Figure S 3: (a) Li^+ number within 2 nm from the electrode for the three scanned reduction rates $6.23/ps$, $0.021/ps$ and $0.007/ps$. (b) The integrated electric charge within 2 nm from the electrode for the three different rates. The charge is initially decreased to negative values due to the reduction reactions. Remarkably, charge neutrality is restored through the migration flux.

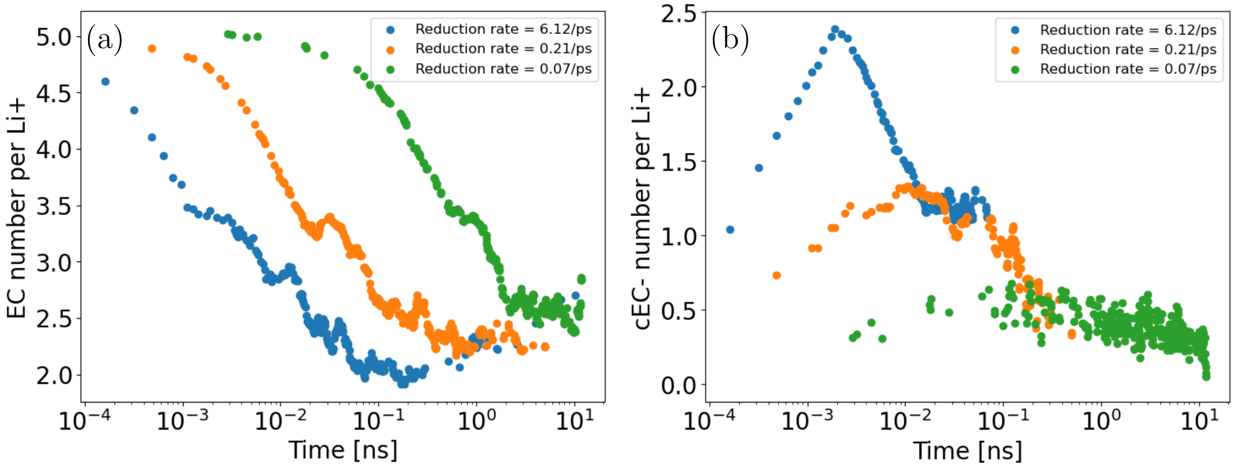

Supplementary Figure S 4: Time dependent coordination numbers of Li^+ (a) The mean number of EC coordinated to Li^+ as a function of time shows that EC reduction is effectively limited by coordination to Li^+. (b) The mean number of coordinated cEC^- to Li^+ shows that complexes including 2 to 3 radicals around Li^+ are stable, thus limiting further coordination of EC to Li^+.

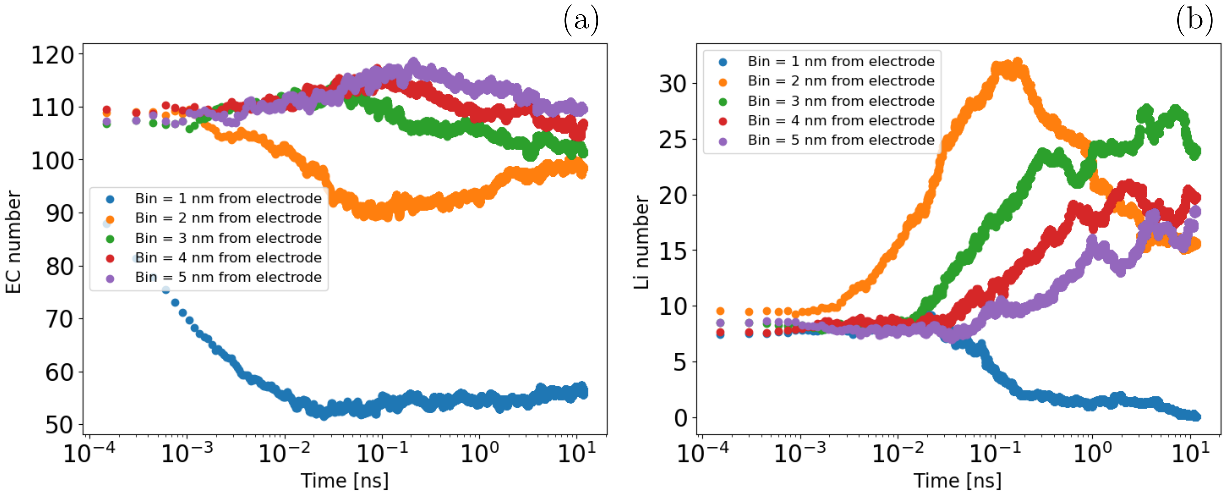

Supplementary Figure S 5: (a) Spatially resolved EC number for bins across the slab direction ($z-$coordinate). The bin width is set to 1 nm and the bins are labeled with respect to the distance from the electrode. The decay in the first bin (the reactive volume) results from the reactions. The fluxes shown on the remaining bins effectively result in a homogeneous EC distribution across slab. (b) Same analysis as in (a) but for the Li^+ number. The flux of Li^+ toward the electrode (increase in bins closest to electrodes) results from EC reduction, and the flux outward the electrode results from the outward flux of Li^+-coordinated products.

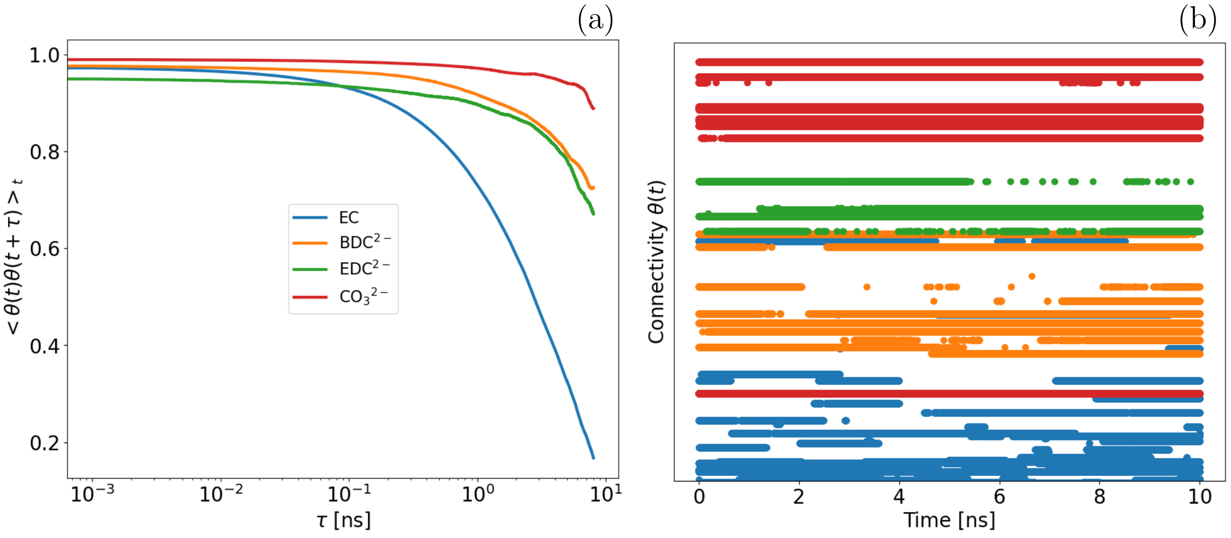

Supplementary Figure S 6: Residence time estimation of Li^+ within the different coordination environments. (a) Autocorrelation function of the connectivity matrix $\theta_{ij}(t)$ as a function of time. A slower decay is identified for CO_3^{2-}, BDC^{2-} and EDC^{2-} as compared to EC. (b) Topological representation of the connectivity matrix $\theta_{ij}(t)$. Each horizential line corresponds to a Li^+, each color represents the molecular labels shown in (a).

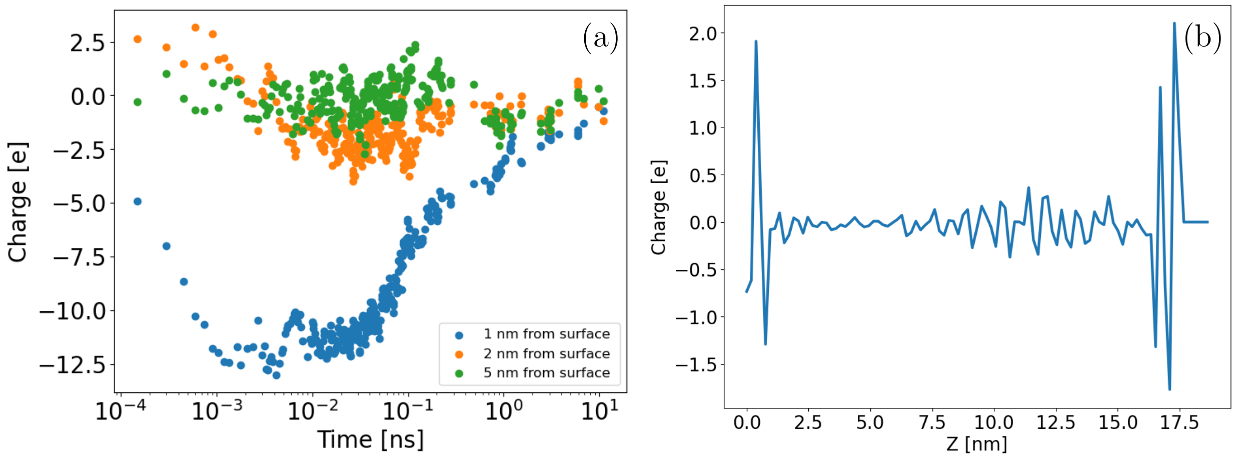

Supplementary Figure S 7: (a) Electric charge for different distances from the electrode as a function of time during the reactive simulations. The representation shows that the deviation from electro-neutrality does not extend further than the first two bins from the electrode. (b) Electric charge profile corresponding to the mass density profile shown in Figure 5 (b) in the main manuscript. The peak corresponds to the surface charge and the bulk region is neutral.

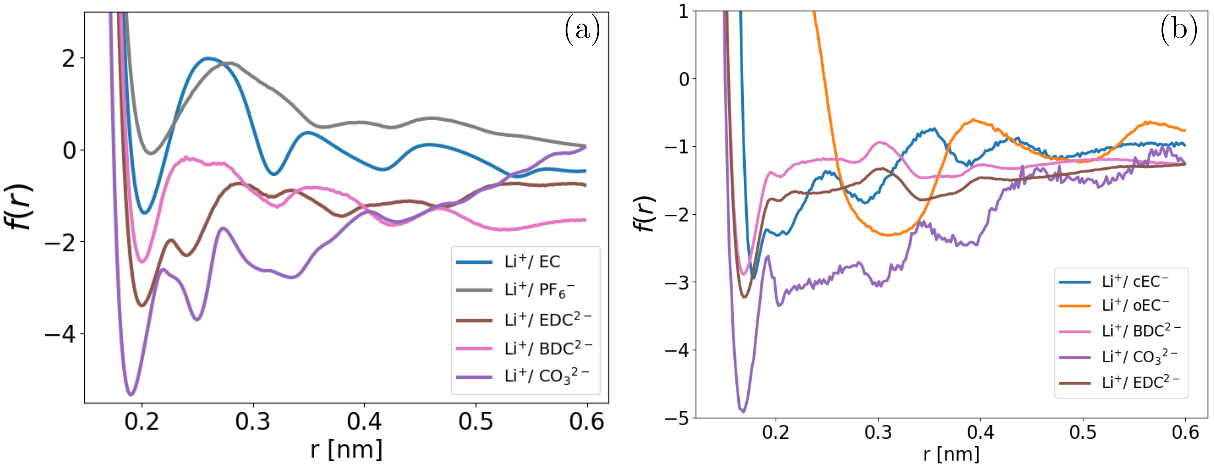

Supplementary Figure S 8: Potential of mean force (PMF) based on the logarithmic radial distribution functions (RDF) of Li^+ with the reaction products CO_3^{2-}, BDC^{2-}, EDC^{2-}, cEC^- and oEC^-. (a) rs@md / OPLS-AA CL+P (b) MDMC / COMPASS 3. The comparison shows that the main features of the binding affinity as obtained from the PMF are equivalent for the two force fields (highest Li^+ binding affinity with CO_3^{2-} and significantly increased first energy barrier).

Supplementary Table S 1: EC reduction rate (theor.) The table summarizes EC reduction rates derived from first-principles simulations in literature. The rates are obtained by extracting the number of reactions normalized by the total number of molecules and the time from each reference.

	System/method	Reaction rate [1/ps]
Kuai, ACS 2022[9]	EC+Lithium / AIMD	1×10^{0}
Leung, PCCP 2010[10]	EC+Lithium / AIMD	1×10^{-1}
Young, ACS 2019[11]	EC+Lithium / AIMD	1×10^{-1}
Leung, JACS 2011[12]	EC+Lithium / AIMD	2×10^{-2}
Islam, JPC 2016[13]	Bulk EC / eReaxff	1×10^{-3}
Takenaka, JPC 2014[14]	EC+Graphite / MDMC	6×10^{-5}
Vargas, JPC 2021[15]	EC+Graphite / MDMC	3×10^{-6}
Hao, JPC 2017[16]	EC+Graphite / Lattice KMC	1×10^{-10}
Li, CPL 2000[17]	EC+continuum solvent / DFT	2×10^{-16}

Supplementary Table S 2: Baseline (exp.) properties of 1M LiPF6 EC carbonate electrolyte

	Method, property	Exp. Value	This work
Kempa, JCS 1958[18]	Denisty	1.32 g/cm^3	1.38
Hayamizu, JCED 2012[19]	Li+ diffusivity / NMR	8×10^{-5} nm^2/ps	5×10^{-5}
Solchenbach, JES 2021[20]	Conductivity / IS	10 mS/cm	–
Solchenbach, JES 2021[20]	Surface capacitance / IS	$1 - 5$ μF/cm^2	3.1
Xu, JPCL 2013[21]	Coordination number / NMR	5.3	4.1
Morita, JSC 1998[22]	Coordination number / Raman	4.1	4.1

Supplementary Table S 3: Activation energy of SEI-conductivity (exp.) The table summarizes activation energies derived from temperature dependent impedance spectroscopy measurements and identified with activation of SEI-conductivity.

	System/method	Activation energy
Steinhauer, EA 2017[23]	1M LiPF6 EC:EMC 3:7	0.25 eV
Xu, JES 2007[21]	1M LiPF6 EC:DMC 3:7	0.75 eV
Solchenbach, JES 2021[20]	1M LiPF6 EC:EMC 3:7	70 kJ/mol
Jow, JES 2012[24]	1M LiPF6 in EC:DMC:MB	67 kJ/mol
llig, JPS 2015[25]	1M LiPF6:LiClO4 1:9 EC:EMC 1:1	75 kJ/mol
Schranzhofer, JPS 2006[26]	1M LiClO4 in PC:AN	0.66 eV
Borodin, JPCC 2013[27]	LiEC and Li2EDC	0.87 eV

Supplementary Table S 4: Activation energy of SEI-growth rate (exp.) The table summarizes activation energies derived from aging rates in temperature dependent cycling experiments of Li-ion cells and identified with SEI growth.

	System	Activation energy
Waldmann, JPS 2014[28]	–	38 kJ/mol
Liaw, JPS 2003[29]	EC:EMC 3:7 1.2 M LiPF6	50 kJ/mol
Deshpande, JES 2012[30]	–	40 kJ/mol
Reniers, JES 2019[31]	–	130 kJ/mol
Schimpe, JES 2018[32]	–	25 kJ/mol
Pinson, JES 2013[33]	EC:DEC 1:1 1 M LiPF6	53 kJ/mol
Plohen, JES 2004[34]	EC:DEC:DMC 1 M LiPF6 + VC	40 kJ/mol

Supplementary Table S 5: EC reduction potential (exp.). The table summarizes reduction potentials derived from cyclic voltammetry measurements and identified with EC reduction.

	System/method	Reduction potential
Nie, JES 2015[35]	3:7 EC:EMC 1.2M LiPF6 / Graphite SFG 6	0.7 V vs Li/Li^+
Novak, JPS 1999[36]	1:1 EC:DMC 1M LiClO4 / Graphite SFG 44	0.8 V vs Li/Li^+
Naji, JPS 1999[37]	EC 1.5M LiClO4 / Graphite UF4	0.9 V vs Li/Li^+
Jeong, JPS 2001[38]	1:1 EC:DEC 1M LiClO4 / Graphite HOPG	1.0 V vs Li/Li^+
Zhang, ACS 2020[39]	3:7 EC:DMC 1M LiPF6 / Graphite HOPG	0.7 V vs Li/Li^+
Yamada, Languimir 2009[40]	1:1 EC:DEC 1M LiClO4 / Graphite HOPG	0.8 V vs Li/Li^+
Zhang, JES 2001[41]	THF 0.1 M LiClO4 EC / Au	1.36 V vs Li/Li^+
Vogel, JES 201[42]5	3:7 EC:DMC 1M LiPF6 / Silicon Si(100)	1.8 V vs Li/Li^+
Salas, JPCC 2012[43]	1:1 EC:DEC 1M LiPF6 / Silicon (a-Si:H)	1.8 V vs Li/Li^+

Supplementary Table S 6: Reaction network [1].

	Reaction	Activation energy [$kcal/mol$]
**I** EC reduction	$EC + e^- + Li^+ \rightarrow cEC^-$	0, 2, 4, 6, 8
**II** cEC ring opening	$cEC^- \rightarrow oEC^-$	2
**III** oEC^- reduction	$oEC^- + e^- + Li^+ \rightarrow CO_3^{2-} + C_2H_4$	0, 2, 4, 6, 8
**IV** oEC^- C-C pairing	$oEC^- + oEC^- \rightarrow BDC^{2-}$	0
**V** cEC^-/oEC^- pairing	$oEC^- + cEC^- \rightarrow EDC^{2-} + C_2H_4$	22.18
**VI** oEC^- C-O pairing	$oEC^- + oEC^- \rightarrow BDC^{2-}$	0

References

[1] Joseph W. Abbott and Felix Hanke. “Kinetically Corrected Monte Carlo–Molecular Dynamics Simulations of Solid Electrolyte Interphase Growth”. In: *Journal of Chemical Theory and Computation* 18.2 (2022). PMID: 35007421, pp. 925–934. DOI: 10.1021/acs.jctc.1c00921. eprint: https://doi.org/10.1021/acs.jctc.1c00921. URL: https://doi.org/10.1021/acs.jctc.1c00921.

[2] Jianmin Tao et al. “Climbing the Density Functional Ladder: Nonempirical Meta–Generalized Gradient Approximation Designed for Molecules and Solids”. In: *Phys. Rev. Lett.* 91 (14 Sept. 2003), p. 146401. DOI: 10.1103/PhysRevLett.91.146401. URL: https://link.aps.org/doi/10.1103/PhysRevLett.91.146401.

[3] R. Ditchfield, W. J. Hehre, and J. A. Pople. “Self-Consistent Molecular-Orbital Methods. IX. An Extended Gaussian-Type Basis for Molecular-Orbital Studies of Organic Molecules”. In: 54.2 (Jan. 1971), pp. 724–728. DOI: 10.1063/1.1674902.

[4] M. J. Frisch et al. *Gaussian˜16 Revision C.01*. Gaussian Inc. Wallingford CT. 2016.

[5] S. Miertuš, E. Scrocco, and J. Tomasi. “Electrostatic interaction of a solute with a continuum. A direct utilizaion of AB initio molecular potentials for the prevision of solvent effects”. In: *Chemical Physics* 55.1 (1981), pp. 117–129. ISSN: 0301-0104. DOI: https://doi.org/10.1016/0301-0104(81)85090-2. URL: https://www.sciencedirect.com/science/article/pii/0301010481850902.

[6] B. Delley. “An all-electron numerical method for solving the local density functional for polyatomic molecules”. In: *The Journal of Chemical Physics* 92.1 (Jan. 1990), pp. 508–517. ISSN: 0021-9606. DOI: 10.1063/1.458452. eprint: https://pubs.aip.org/aip/jcp/article-pdf/92/1/508/11172635/508_1_online.pdf. URL: https://doi.org/10.1063/1.458452.

[7] Abbas Farazdel et al. "Electric-field induced intramolecular electron transfer in spiro .pi.-electron systems and their suitability as molecular electronic devices. A theoretical study". In: *Journal of the American Chemical Society* 112.11 (1990), pp. 4206–4214. DOI: 10.1021/ja00167a016. eprint: https://doi.org/10.1021/ja00167a016. URL: https://doi.org/10.1021/ja00167a016.

[8] E. Aprà et al. "NWChem: Past, present, and future". In: *The Journal of Chemical Physics* 152.18 (2020), p. 184102. DOI: 10.1063/5.0004997.

[9] Dacheng Kuai and Perla B. Balbuena. "Solvent Degradation and Polymerization in the Li-Metal Battery: Organic-Phase Formation in Solid-Electrolyte Interphases". In: *ACS Applied Materials & Interfaces* 14.2 (2022). PMID: 34994191, pp. 2817–2824. DOI: 10.1021/acsami.1c20487. eprint: https://doi.org/10.1021/acsami.1c20487. URL: https://doi.org/10.1021/acsami.1c20487.

[10] Kevin Leung and Joanne L. Budzien. "Ab initio molecular dynamics simulations of the initial stages of solid–electrolyte interphase formation on lithium ion battery graphitic anodes". In: *Phys. Chem. Chem. Phys.* 12 (25 2010), pp. 6583–6586. DOI: 10.1039/B925853A. URL: http://dx.doi.org/10.1039/B925853A.

[11] Joshua Young et al. "Comparative Study of Ethylene Carbonate-Based Electrolyte Decomposition at Li, Ca, and Al Anode Interfaces". In: *ACS Applied Energy Materials* 2.3 (2019), pp. 1676–1684. DOI: 10.1021/acsaem.8b01707. eprint: https://doi.org/10.1021/acsaem.8b01707. URL: https://doi.org/10.1021/acsaem.8b01707.

[12] Kevin Leung et al. "Using Atomic Layer Deposition to Hinder Solvent Decomposition in Lithium Ion Batteries: First-Principles Modeling and Experimental Studies". In: *Journal of the American Chemical Society* 133.37 (2011). PMID: 21797223, pp. 14741–14754. DOI: 10.1021/ja205119g. eprint: https://doi.org/10.1021/ja205119g. URL: https://doi.org/10.1021/ja205119g.

[13] Md Mahbubul Islam and Adri C. T. van Duin. “Reductive Decomposition Reactions of Ethylene Carbonate by Explicit Electron Transfer from Lithium: An eReaxFF Molecular Dynamics Study”. In: *The Journal of Physical Chemistry C* 120.48 (2016), pp. 27128–27134. DOI: 10.1021/acs.jpcc.6b08688. eprint: https://doi.org/10.1021/acs.jpcc.6b08688. URL: https://doi.org/10.1021/acs.jpcc.6b08688.

[14] Norio Takenaka et al. “On Electrolyte-Dependent Formation of Solid Electrolyte Interphase Film in Lithium-Ion Batteries: Strong Sensitivity to Small Structural Difference of Electrolyte Molecules”. In: *The Journal of Physical Chemistry C* 118.20 (2014), pp. 10874–10882. DOI: 10.1021/jp5018696. eprint: https://doi.org/10.1021/jp5018696. URL: https://doi.org/10.1021/jp5018696.

[15] Lorena Alzate-Vargas et al. “Insight into SEI Growth in Li-Ion Batteries using Molecular Dynamics and Accelerated Chemical Reactions”. In: *The Journal of Physical Chemistry C* 125.34 (2021), pp. 18588–18596. DOI: 10.1021/acs.jpcc.1c04149. eprint: https://doi.org/10.1021/acs.jpcc.1c04149. URL: https://doi.org/10.1021/acs.jpcc.1c04149.

[16] Feng Hao et al. “Mesoscale Elucidation of Solid Electrolyte Interphase Layer Formation in Li-Ion Battery Anode”. In: *The Journal of Physical Chemistry C* 121.47 (2017), pp. 26233–26240. DOI: 10.1021/acs.jpcc.7b09465. eprint: https://doi.org/10.1021/acs.jpcc.7b09465. URL: https://doi.org/10.1021/acs.jpcc.7b09465.

[17] Tao Li and Perla B. Balbuena. “Theoretical studies of the reduction of ethylene carbonate”. In: *Chemical Physics Letters* 317.3 (2000), pp. 421–429. ISSN: 0009-2614. DOI: https://doi.org/10.1016/S0009-2614(99)01374-3. URL: https://www.sciencedirect.com/science/article/pii/S0009261499013743.

[18] R. Kempa and W. H. Lee. “392. The dipole moments of some cyclic carbonates”. In: *J. Chem. Soc.* (0 1958), pp. 1936–1938. DOI: 10.1039/JR9580001936. URL: http://dx.doi.org/10.1039/JR9580001936.

[19] Kikuko Hayamizu. "Temperature Dependence of Self-Diffusion Coefficients of Ions and Solvents in Ethylene Carbonate, Propylene Carbonate, and Diethyl Carbonate Single Solutions and Ethylene Carbonate + Diethyl Carbonate Binary Solutions of LiPF6 Studied by NMR". In: *Journal of Chemical & Engineering Data* 57.7 (2012), pp. 2012–2017. DOI: 10.1021/je3003089. eprint: https://doi.org/10.1021/je3003089. URL: https://doi.org/10.1021/je3003089.

[20] Sophie Solchenbach et al. "Monitoring SEI Formation on Graphite Electrodes in Lithium-Ion Cells by Impedance Spectroscopy". In: *Journal of The Electrochemical Society* 168.11 (Nov. 2021), p. 110503. DOI: 10.1149/1945-7111/ac3158. URL: https://dx.doi.org/10.1149/1945-7111/ac3158.

[21] Kang Xu. ""Charge-Transfer" Process at Graphite/Electrolyte Interface and the Solvation Sheath Structure of Li+ in Nonaqueous Electrolytes". In: *Journal of The Electrochemical Society* 154.3 (Jan. 2007), A162. DOI: 10.1149/1.2409866. URL: https://dx.doi.org/10.1149/1.2409866.

[22] Masayuki Morita et al. "A Raman spectroscopic study of organic electrolyte solutions based on binary solvent systems of ethylene carbonate with low viscosity solvents which dissolve different lithium salts". In: *J. Chem. Soc., Faraday Trans.* 94 (23 1998), pp. 3451–3456. DOI: 10.1039/A806278A. URL: http://dx.doi.org/10.1039/A806278A.

[23] Miriam Steinhauer et al. "Investigation of the Solid Electrolyte Interphase Formation in Lithium-Ion Batteries with Electrochemical Impedance Spectroscopy". In: *Electrochimica Acta* 228 (Jan. 2017), pp. 652–658. URL: https://elib.dlr.de/111175/.

[24] T. Richard Jow, Michelle B. Marx, and Jan L. Allen. "Distinguishing Li+ Charge Transfer Kinetics at NCA/Electrolyte and Graphite/Electrolyte Interfaces, and NCA/Electrolyte and LFP/Electrolyte Interfaces in Li-Ion Cells". In: *Journal of The Electrochemical So-

ciety 159.5 (Mar. 2012), A604. DOI: 10.1149/2.079205jes. URL: https://dx.doi.org/10.1149/2.079205jes.

[25] J. Illig et al. "Modeling graphite anodes with serial and transmission line models". In: *Journal of Power Sources* 282 (2015), pp. 335–347. ISSN: 0378-7753. DOI: https://doi.org/10.1016/j.jpowsour.2015.02.038. URL: https://www.sciencedirect.com/science/article/pii/S037877531500261X.

[26] H. Schranzhofer et al. "Electrochemical impedance spectroscopy study of the SEI formation on graphite and metal electrodes". In: *Journal of Power Sources* 153.2 (2006). Selected papers presented at the 2004 Meeting of the International Battery Association, pp. 391–395. ISSN: 0378-7753. DOI: https://doi.org/10.1016/j.jpowsour.2005.05.034. URL: https://www.sciencedirect.com/science/article/pii/S0378775305007342.

[27] Oleg Borodin et al. "Molecular Dynamics Simulations and Experimental Study of Lithium Ion Transport in Dilithium Ethylene Dicarbonate". In: *The Journal of Physical Chemistry C* 117.15 (2013), pp. 7433–7444. DOI: 10.1021/jp4000494. eprint: https://doi.org/10.1021/jp4000494. URL: https://doi.org/10.1021/jp4000494.

[28] Thomas Waldmann et al. "Temperature dependent ageing mechanisms in Lithium-ion batteries – A Post-Mortem study". In: *Journal of Power Sources* 262 (2014), pp. 129–135. ISSN: 0378-7753. DOI: https://doi.org/10.1016/j.jpowsour.2014.03.112. URL: https://www.sciencedirect.com/science/article/pii/S0378775314004352.

[29] Bor Yann Liaw et al. "Correlation of Arrhenius behaviors in power and capacity fades with cell impedance and heat generation in cylindrical lithium-ion cells". In: *Journal of Power Sources* 119-121 (2003). Selected papers presented at the 11th International Meeting on Lithium Batteries, pp. 874–886. ISSN: 0378-7753. DOI: https://doi.

org/10.1016/S0378-7753(03)00196-4. URL: https://www.sciencedirect.com/science/article/pii/S0378775303001964.

[30] Rutooj Deshpande et al. "Battery Cycle Life Prediction with Coupled Chemical Degradation and Fatigue Mechanics". In: *Journal of The Electrochemical Society* 159.10 (Aug. 2012), A1730. DOI: 10.1149/2.049210jes. URL: https://dx.doi.org/10.1149/2.049210jes.

[31] Jorn M. Reniers, Grietus Mulder, and David A. Howey. "Review and Performance Comparison of Mechanical-Chemical Degradation Models for Lithium-Ion Batteries". In: *Journal of The Electrochemical Society* 166.14 (Sept. 2019), A3189. DOI: 10.1149/2.0281914jes. URL: https://dx.doi.org/10.1149/2.0281914jes.

[32] M. Schimpe et al. "Comprehensive Modeling of Temperature-Dependent Degradation Mechanisms in Lithium Iron Phosphate Batteries". In: *Journal of The Electrochemical Society* 165.2 (Jan. 2018), A181. DOI: 10.1149/2.1181714jes. URL: https://dx.doi.org/10.1149/2.1181714jes.

[33] Matthew B. Pinson and Martin Z. Bazant. "Theory of SEI Formation in Rechargeable Batteries: Capacity Fade, Accelerated Aging and Lifetime Prediction". In: *Journal of The Electrochemical Society* 160.2 (Dec. 2012), A243. DOI: 10.1149/2.044302jes. URL: https://dx.doi.org/10.1149/2.044302jes.

[34] Harry J. Ploehn, Premanand Ramadass, and Ralph E. White. "Solvent Diffusion Model for Aging of Lithium-Ion Battery Cells". In: *Journal of The Electrochemical Society* 151.3 (Feb. 2004), A456. DOI: 10.1149/1.1644601. URL: https://dx.doi.org/10.1149/1.1644601.

[35] Mengyun Nie et al. "Effect of Vinylene Carbonate and Fluoroethylene Carbonate on SEI Formation on Graphitic Anodes in Li-Ion Batteries". In: *Journal of The Electrochemical Society* 162.13 (July 2015), A7008. DOI: 10.1149/2.0021513jes. URL: https://dx.doi.org/10.1149/2.0021513jes.

[36] Petr Novák et al. "In situ investigation of the interaction between graphite and electrolyte solutions". In: *Journal of Power Sources* 81-82 (1999), pp. 212–216. ISSN: 0378-7753. DOI: https://doi.org/10.1016/S0378-7753(99)00119-6. URL: https://www.sciencedirect.com/science/article/pii/S0378775399001196.

[37] A. Naji et al. "Electroreduction of graphite in LiClO4-ethylene carbonate electrolyte. Characterization of the passivating layer by transmission electron microscopy and Fourier-transform infrared spectroscopy". In: *Journal of Power Sources* 63.1 (1996), pp. 33–39. ISSN: 0378-7753. DOI: https://doi.org/10.1016/S0378-7753(96)02439-1. URL: https://www.sciencedirect.com/science/article/pii/S0378775396024391.

[38] Soon-Ki Jeong et al. "Surface Film Formation on Graphite Negative Electrode in Lithium-Ion Batteries: AFM Study in an Ethylene Carbonate-Based Solution". In: *Journal of The Electrochemical Society* 148.9 (Aug. 2001), A989. DOI: 10.1149/1.1387981. URL: https://dx.doi.org/10.1149/1.1387981.

[39] Zhenyu Zhang et al. "Operando Electrochemical Atomic Force Microscopy of Solid–Electrolyte Interphase Formation on Graphite Anodes: The Evolution of SEI Morphology and Mechanical Properties". In: *ACS Applied Materials & Interfaces* 12.31 (2020). PMID: 32657567, pp. 35132–35141. DOI: 10.1021/acsami.0c11190. eprint: https://doi.org/10.1021/acsami.0c11190. URL: https://doi.org/10.1021/acsami.0c11190.

[40] Yuki yamada2009 et al. "Kinetics of Lithium Ion Transfer at the Interface between Graphite and Liquid Electrolytes: Effects of Solvent and Surface Film". In: *Langmuir* 25.21 (2009). PMID: 19856995, pp. 12766–12770. DOI: 10.1021/la901829v. eprint: https://doi.org/10.1021/la901829v. URL: https://doi.org/10.1021/la901829v.

[41] Xuerong Zhang et al. "Electrochemical and Infrared Studies of the Reduction of Organic Carbonates". In: *Journal of The Electrochemical Society* 148.12 (Nov. 2001), A1341. DOI: 10.1149/1.1415547. URL: https://dx.doi.org/10.1149/1.1415547.

[42] Ulrike S. Vogl et al. "The Mechanism of SEI Formation on a Single Crystal Si(100) Electrode". In: *Journal of The Electrochemical Society* 162.4 (Jan. 2015), A603. DOI: 10.1149/2.0391504jes. URL: https://dx.doi.org/10.1149/2.0391504jes.

[43] David E. Arreaga-Salas et al. "Progression of Solid Electrolyte Interphase Formation on Hydrogenated Amorphous Silicon Anodes for Lithium-Ion Batteries". In: *The Journal of Physical Chemistry C* 116.16 (2012), pp. 9072–9077. DOI: 10.1021/jp300787p. eprint: https://doi.org/10.1021/jp300787p. URL: https://doi.org/10.1021/jp300787p.
